# Receptor-mediated drug delivery of bispecific therapeutic antibodies through the blood-brain barrier

**DOI:** 10.3389/fddev.2023.1227816

**Published:** 2023-07-10

**Authors:** William M. Pardridge

**Affiliations:** University of California, Los Angeles, Los Angeles, CA, United States

**Keywords:** receptor-mediated transcytosis, insulin receptor, transferrin receptor, monoclonal antibody, Alzheimer’s disease, Parkinson’s disease

## Abstract

Therapeutic antibody drug development is a rapidly growing sector of the pharmaceutical industry. However, antibody drug development for the brain is a technical challenge, and therapeutic antibodies for the central nervous system account for ∼3% of all such agents. The principal obstacle to antibody drug development for brain or spinal cord is the lack of transport of large molecule biologics across the blood-brain barrier (BBB). Therapeutic antibodies can be made transportable through the blood-brain barrier by the re-engineering of the therapeutic antibody as a BBB-penetrating bispecific antibody (BSA). One arm of the BSA is the therapeutic antibody and the other arm of the BSA is a transporting antibody. The transporting antibody targets an exofacial epitope on a BBB receptor, and this enables receptor-mediated transcytosis (RMT) of the BSA across the BBB. Following BBB transport, the therapeutic antibody then engages the target receptor in brain. RMT systems at the BBB that are potential conduits to the brain include the insulin receptor (IR), the transferrin receptor (TfR), the insulin-like growth factor receptor (IGFR) and the leptin receptor. Therapeutic antibodies have been re-engineered as BSAs that target the insulin receptor, TfR, or IGFR RMT systems at the BBB for the treatment of Alzheimer’s disease and Parkinson’s disease.

## 1 Introduction

Therapeutic monoclonal antibodies (MAb) are the fastest growing sector of the pharmaceutical industry (Lu et al, 2020). As of June 2022, a total of 162 therapeutic antibodies had been approved world-wide (Lyu et al, 2022). The majority of the approved therapeutic antibodies treat cancer or immune-related diseases, and only ∼3% of approved therapeutic antibodies treat the central nervous system (CNS) (Lyu et al, 2022). The development of therapeutic antibodies for the CNS is made difficult by the presence of the blood-brain barrier (BBB), and to the lack of therapeutic antibody transport across the BBB. The BBB is localized to the endothelial wall of the capillaries perfusing the brain. Therapeutic antibodies can be made transportable through the BBB following the re-engineering of the antibody as a BBB-penetrating bispecific antibody (BSA). One antibody arm of the BSA is the therapeutic antibody and the other antibody arm of the BSA is an antibody that penetrates the BBB via receptor-mediated transcytosis (RMT). The RMT antibody acts as a molecular Trojan horse to ferry the therapeutic antibody across the BBB to reach target sites within the CNS. The RMT antibody targets certain endogenous peptide receptors on the BBB that serve to mediate the uptake by brain of circulating peptides, such as insulin, transferrin (Tf), the insulin-like growth factors (IGF), or leptin. The expression of an insulin receptor (INSR or IR), a Tf receptor (TfR), an IGF receptor (IGFR), or a leptin receptor (LEPR) on the human BBB was identified over 25 years ago with capillaries isolated from human brain (Pardridge et al, 1985; Pardridge et al, 1987a; Duffy et al, 1988; Golden et al, 1997). These brain endothelial receptors enable the RMT of the cognate peptides through the BBB as discussed below in Section 3.1.

The need to re-engineer a therapeutic antibody for the CNS as a BBB-penetrating BSA might be questioned owing to a) the fact that several therapeutic antibodies are already FDA approved drugs for the brain, and b) there is purported to be evidence that therapeutic antibodies cross the BBB. In Section 2 below, the view will be presented that the current FDA approved therapeutic antibodies either do not have a site of action within the CNS, or do not cross the BBB in the absence of BBB disruption; Section 2 will also review current methodologies used to assess BBB transport of therapeutic antibodies, and will emphasize how the misinterpretation of these methods can lead to the conclusion that a therapeutic antibody enters the parenchyma of brain from blood when, in fact, the antibody does not cross the BBB. In Section 3.3 below, the engineering of BBB-penetrating BSAs will be reviewed for therapeutic antibodies that target Abeta amyloid, beta secretase 1 (BACE1), or triggering receptor for myeloid cells 2 (TREM2), for the treatment of Alzheimer’s disease (AD), and therapeutic antibodies that target α-synuclein (SYN), or tropomyosin receptor kinase B (trkB), for the treatment of Parkinson’s disease (PD).

## 2 Development of therapeutic antibodies for the CNS without blood-brain barrier drug delivery technology

### 2.1 FDA approved therapeutic antibodies for the CNS

#### 2.1.1 Therapeutic antibodies for multiple sclerosis

Several therapeutic antibodies have been approved for treatment of relapsing multiple sclerosis (MS) since natalizumab was introduced in 2004 (Cadavid et al, 2013). However, none of these approved antibodies for MS has a site of action within the CNS beyond the BBB, as all work on the immune system within the blood compartment. Natalizumab binds α-4 integrins, and suppresses the uptake of activated lymphocytes by brain via inhibition of lymphocyte adhesion to the blood side of the brain endothelium (Engelhardt and Coisne, 2011). Alemtuzumab suppresses lymphocyte function in blood by binding the CD52 receptor on lymphocytes, which triggers Fc receptor (FcR)-mediated effector function to causes antibody dependent cell-mediated cytotoxicity (ADCC) (Voge and Alvarez, 2019). Similar to rituximab, ocrelizumab targets the CD20 receptor on lymphocytes (Voge and Alvarez, 2019), but is engineered with multiple amino acid substitutions in the Fc region that selectively enhance complement mediated cytotoxicity (CDC) of lymphocytes in blood (Saxena and Wu, 2016). Ublituximab suppresses blood lymphocytes via glyco-engineering of the Fc region of the antibody (Voge and Alvarez, 2019; Lee, 2023). This antibody is expressed in a specific host cell line that produces a carbohydrate region deficient in fucose (Pereira et al, 2018). Antibodies deficient in core fucose have increased affinity for the FcγRIIIa (Ferrara et al, 2011), which results in increased ADCC (Kanda et al, 2006). Therapeutic antibodies that target receptors within the CNS and behind the BBB are not expected to be effective in the treatment of MS, owing to lack of antibody transport across the BBB. This is illustrated in the failed clinical trial of opicinumab in MS, even at the very high dose of 100 mg/kg (Kramer and Wiendl, 2022). This antibody inhibits leucine-rich repeat and immunoglobulin-like domain-containing Nogo receptor-interacting protein 1 (LINGO1), a protein that is selectively expressed on oligodendrocytes and neurons in brain (Kramer and Wiendl, 2022), which are behind the BBB.

#### 2.1.2 Therapeutic antibodies for brain cancer

Bevacizumab suppresses tumor blood vessel growth by binding vascular endothelial growth factor (VEGF) in blood, and was approved for second line treatment of recurrent glioblastoma multiforme (GBM) in 2009 (Han et al, 2014). Bevacizumab does not cross the intact BBB (Liu et al, 2016), and works via the sequestration of VEGF in the brain capillary compartment, which suppresses tumor angiogenesis (Keunen et al, 2011). The VEGF receptor 1, also known as Flt-1, is expressed at the brain microvascular endothelium, as demonstrated with a BBB genomics study (Li et al, 2001). Suppression of tumor angiogenesis, which produces regions of hypoxia, can have a paradoxical effect to enhance tumor growth (Keunen et al, 2011). Bevacizumab treatment does not increase the overall survival in GBM (Fu et al, 2016), and confers no benefit over temozolomide therapy (Han et al, 2014). The BBB is disrupted in certain regions of GBM (Sarkaria et al, 2018). However, significant parts of a GBM tumor are perfused by capillaries with an intact BBB, and these treatment-resistant regions of the GBM give rise to tumor recurrence (Sarkaria et al, 2018). With respect to metastatic cancer to brain, up to 50% of HER2 positive breast cancer results in brain metastasis (Zimmer et al, 2022). Trastuzumab is a primary treatment for HER2-positive breast cancer, but trastuzumab does not cross the BBB (Kinoshita et al, 2006). The BBB is generally intact in breast cancer metastasis to brain (Bailleux et al, 2021), and trastuzumab therapy does not effectively treat breast cancer metastases to brain (Bria et al, 2008).

#### 2.1.3 Therapeutic antibodies for Alzheimer’s disease

##### 2.1.3.1 Anti-Abeta amyloid therapeutic antibodies in Alzheimer’s disease

The dementia of AD correlates with the deposition in brain of amyloid plaque (Cummings and Cotman, 1995; Naslund et al, 2000). The amyloid plaque that forms around brain blood vessels and in brain extracellular space in AD is derived from a peptide of 40–43 amino acids in length (Glenner and Wong, 1984; Masters et al, 1985), known as the Aβ amyloid peptide. Amino acid composition analysis of the Aβ amyloid isolated from meningeal vessels (Glenner and Wong, 1984), neuritic plaque (Masters et al, 1985), or intra-cortical parenchymal vessels (Pardridge et al, 1987b) of AD brain all show a single threonine (Thr) residue, and the only Thr in the Aβ peptide is at position 43 (Kang et al, 1987). Anti-Aβ amyloid antibodies (AAA) disaggregate Aβ fibrils *in vitro* (Solomon et al, 1997). The intra-cerebral injection of an early AAA, the 3D6 antibody, into the brain of AD transgenic mice resulted in the clearance of amyloid plaque at the injection site in brain, which was followed by local repair of dystrophic neurites (Lombardo et al, 2003). A humanized version of the murine 3D6 antibody, bapineuzumab, entered into clinical trials for AD, where the AAA was administered by intravenous IV) infusion every 3 months at an infusion dose (ID) of 0.5, 1, or 2 mg/kg (Salloway et al, 2014). The ID of 2 mg/kg was discontinued owing to the presence of amyloid relating imaging abnormalities-edema (ARIA-E) observed with magnetic resonance imaging (MRI). As discussed below, ARIA-E represents AAA induced brain edema associated with BBB disruption, and is observed following the administration of an AAA in both AD patients (Cogswell et al, 2022; Nehra et al, 2022) and AD transgenic mice (Blockx et al, 2016). The brain uptake of the murine precursor of bapineuzumab in the mouse (Bard et al, 2012) is very low, 0.07% ID/Gram, which is consistent with entrapment of the antibody in the blood volume of brain (Sumbria et al, 2013a). It is proposed that the lack of transport of bapineuzumab through an intact BBB played a pivotal role in the bapineuzumab clinical trial failure (Pardridge, 2019).

**FIGURE 1:**
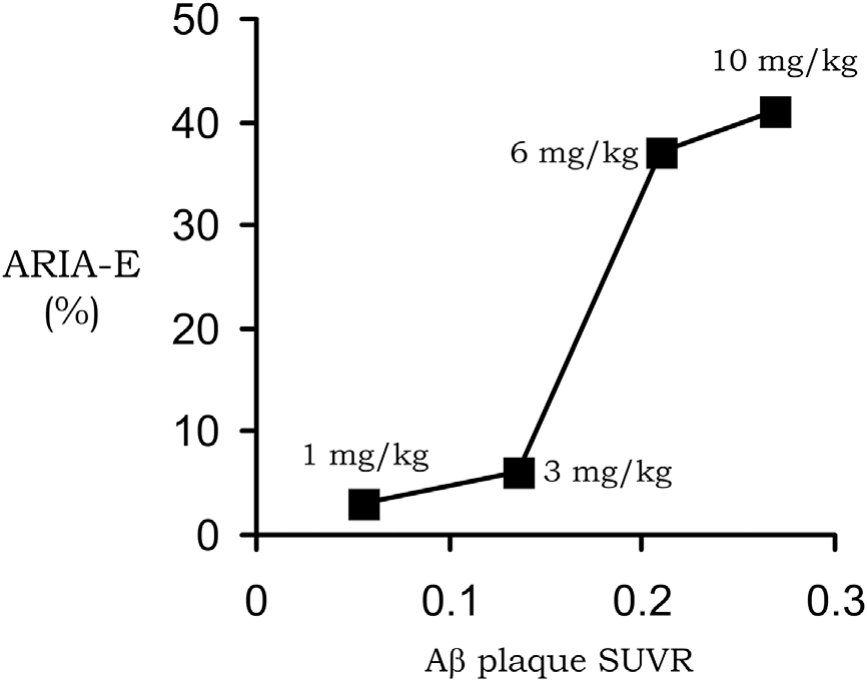
Near linear correlation between ARIA-E and plaque reduction in AD subjects treated with 1–10 mg/kg doses of aducanumab. Reproduced with permission from Pardridge (2019).

Despite the failure of the bapineuzumab trial, over a dozen AAAs entered into clinical trials for the treatment of AD (Pardridge, 2020). One of these antibodies, aducanumab, resulted in a dose-dependent reduction in the brain amyloid plaque, as quantified with florbetapir PET scanning (Sevigny et al, 2016). In 2021, aducanumab was the first AAA approved by the FDA for the treatment of AD, albeit amid controversy (Liu and Howard, 2021). Aducanumab as a treatment for AD was rejected by the healthcare community (Bauchner and Alexander, 2022). Aducanumab was approved despite the lack of evidence that this therapeutic antibody crossed the intact BBB. Aducanumab was said to cross the BBB (Sevigny et al, 2016), but the negligible brain uptake of aducanumab was demonstrated to represent entrapment of the antibody in the brain blood volume (Pardridge, 2019), as discussed below in Section 2.2.2. The reduction of the amyloid plaque in brain caused by aducanumab treatment of patients with AD (Sevigny et al, 2016) indicated the antibody did gain access to brain via transport across the BBB. The mechanism by which aducanumab enters the brain in AD was suggested by the finding that aducanumab resulted in ARIA of the brain (Sevigny et al, 2016), which is indicative of BBB disruption (Blockx et al, 2016; Cogswell et al, 2022; Nehra et al, 2022). An analysis of the aducanumab reduction in brain amyloid and induction of ARIA showed there was a linear relationship between these parameters (Pardridge, 2019), as shown in Figure 1. This near linear relationship between amyloid reduction and ARIA development suggests aducanumab enters the brain following antibody treatment induced BBB breakdown leading to ARIA. This hypothesis was subsequently confirmed by the finding of a correlation in AAA clinical trials between brain amyloid plaque reduction and induction of ARIA-E for aducanumab, donanemab, lecanemab, and gantenerumab (Wang et al, 2022). In support of these findings, cerenezumab, which does not reduce amyloid plaque in brain in AD, also does not cause ARIA-E (Ostrowitzki et al, 2022). ARIA-E (edema) and ARIA-H (hemorrhage) is caused by the leakage into brain of plasma and red blood cells, respectively (Cogswell et al, 2022). The leakage of red blood cells is associated with the parallel leakage of plasma so that ARIA-E co-exists with ARIA-H (Cogswell et al, 2022). The development of ARIA-E, or ARIA-H, and BBB disruption following the administration of an AAA to AD subjects is analogous to the cerebral micro-hemorrhage observed in AD mice following the treatment with high doses of the AAA (Wilcock and Colton, 2009). ARIA is also detected by MRI of brain following chronic administration of the 3D6 AAA in PDAPP transgenic mice (Blockx et al, 2016). In the absence of AAA treatment, the BBB is intact in AD, even to small molecule imaging agents, as determined by PET (Schlageter et al, 1987), contrast computed tomography (Caserta et al, 1998), and contrast MRI (Starr et al, 2009).

In 2023, a second AAA, lecanemab, received accelerated approval from the FDA for AD (Cummings et al, 2023). The primary clinical endpoint in the lecanemab trial was the Clinical Dementia Rating-Sum of Boxes (CDR-SB). The CDR-SB scale encompasses 18 points, and lecanemab treatment resulted in an improvement of 0.45 points (Van Dyck et al, 2023). This is a fairly meager pharmacologic effect, as a clinical meaningful change in the CDR-SB is at least one to two points (Andrews et al, 2019). Lecanemab treatment causes both ARIA-E and ARIA-H, and the warning of these side effects is part of the drug label (Mahase, 2023). The mechanism of how lecanemab crosses the non-disrupted BBB to gain access to amyloid plaque is not discussed in the lecanemab drug development process (Cummings et al, 2023). Lecanemab, also known as BAN2401, is a humanized version of the murine mAb158. The brain uptake in the mouse of mAb158 is very low, 0.028% ID/Gram (Hultqvist et al, 2017), which is consistent with entrapment of the antibody within the plasma volume of brain without BBB transport. The mAb158 has been re-engineered as a BBB-penetrating BSA as discussed below in Section 3.3.1.

Donanemab is another AAA for AD, which is near FDA approval (Gueorguieva et al, 2023). Donanemab is a humanized version of the pE3 MAb. pE3 is Aβ^3-42^, where the amino terminal Asp^1^-Ala^2^ is cleaved producing an N-terminal Glu^3^, which cyclizes to the pyroglutamate form (Demattos et al, 2012). The primary endpoint in the donanemab trial for AD was the sponsor’s Integrated AD Rating Scale (iADRS), where the CDR-SB was the secondary endpoint (Mintun et al, 2021). Reduction in brain amyloid was only observed at the high dose, 10 mg/kg, of donanemab administered monthly for 72 weeks (Lowe et al, 2021). This treatment produced a very high plasma concentration of the AAA of ∼1 uM (Gueorguieva et al, 2023), which is nearly 1% of all IgG in plasma. The effect of donanemab treatment on the CDR-SB is modest, 0.7 points on the 18-point scale, and treatment with this AAA causes ARIA-E/ARIA-H in 31% of subjects (Travis, 2023). These results for donanemab are similar to the lecanemab and aducanumab trials discussed above. Medicare coverage for these AAAs for AD is restricted (Brockmann et al, 2023), and the Centers for Medicare and Medicaid Services requires that prescribing doctors must participate in a health agency registry, a limitation generally used by Medicare for medical devices, not pharmaceuticals (Steenhuysen, 2023).

##### 2.1.3.2 Anti-tau therapeutic antibodies in Alzheimer’s disease

Intracellular insoluble aggregates accumulate in neurons in AD to form neurofibrillary tangles (Kuchibhotla et al, 2014), which are derived from abnormal processing of the tau microtubule protein. Therapeutic antibodies against the tau protein monomer or fibril, with or without phosphorylation, are currently in clinical trials (Pardridge, 2020; Ji and Sugurdsson, 2021). Anti-tau antibodies include semorinemab, and ABBV-8E12, also known as tilavonemab, and these antibodies have been recently reported to have no efficacy in AD (Teng et al, 2022; Florian et al, 2023). Failure of the early tau antibody trials in AD is said to be reminiscent of the failures of the early Abeta amyloid antibody trials in AD (Imbimbo et al, 2023). The difference is that anti-tau antibodies, unlike anti-Abeta amyloid antibodies, do not cause ARIA-E, and do not disrupt the BBB, and thus have no mechanism to gain access to brain tissue from blood.

In summary, the therapeutic antibodies that have been FDA approved to date for the CNS either do not have a site of action within the CNS, as in the case of the antibodies for MS or glioma, or gain access to brain via BBB disruption, as in the case of the anti-amyloid antibodies for AD. Nevertheless, there are numerous other therapeutic antibodies currently in clinical trials for CNS disease, and the rationale for these trials is that therapeutic antibodies have a low, but pharmacologically significant, transport across the BBB. This rationale will be examined in the following section, where the methodologies used to assess transport of therapeutic antibodies into brain are reviewed.

### 2.2 Methods used to assess transport of therapeutic antibodies through the blood-brain barrier

#### 2.2.1 Cerebrospinal fluid

**FIGURE 2:**
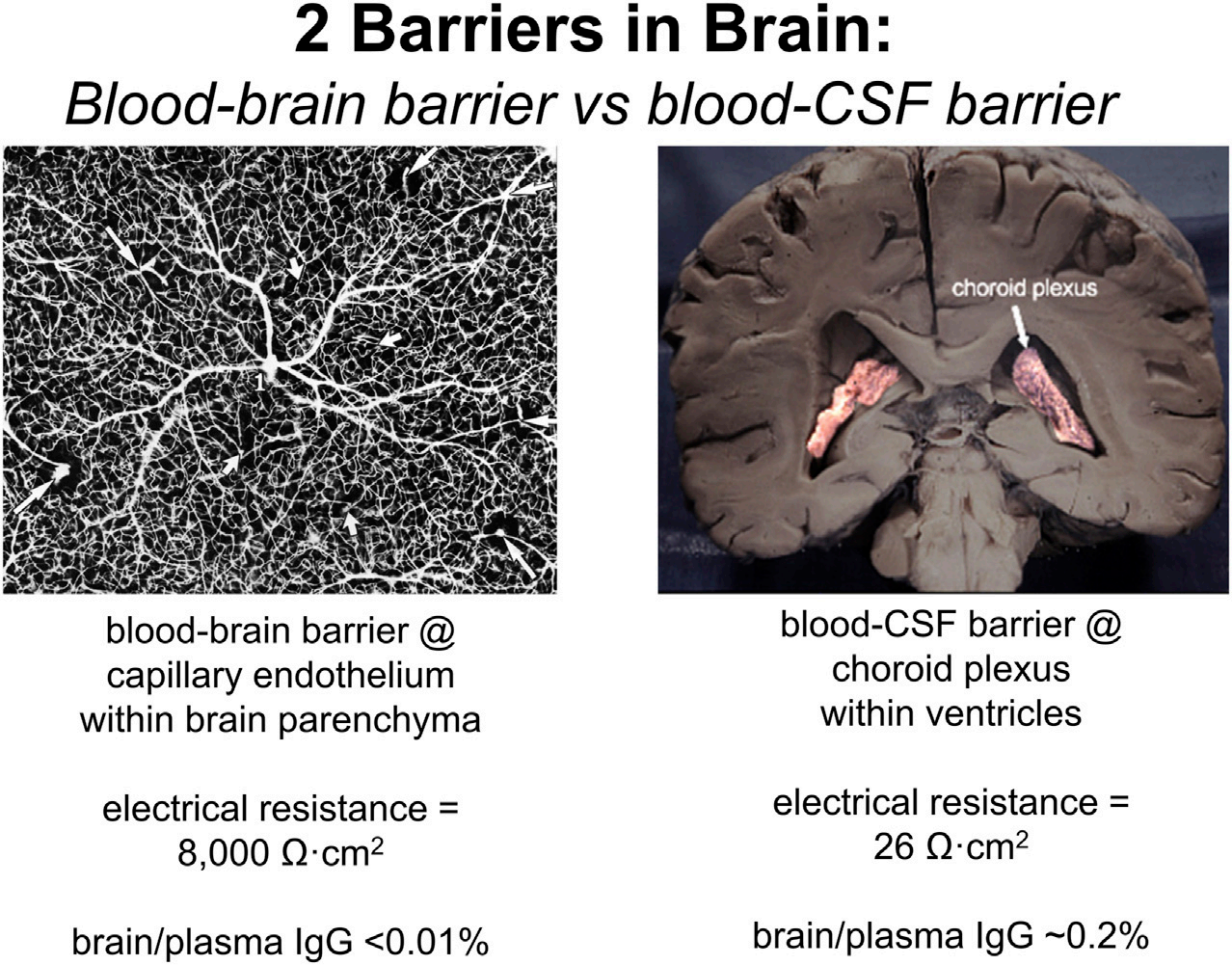
The 2 barriers in brain are the blood-brain barrier (BBB) and the blood-cerebrospinal fluid (CSF) barrier. (Left panel) The BBB is formed by the endothelium of capillaries that perfuse brain parenchyma as shown by the inverted image of an India ink infusion of the human brain. The brain endothelia are joined by tight junctions with high electrical resistance of 8,000 Ω cm^2^, and the brain/plasma ratio of IgG is very low, <0.01%. (Right panel) The blood-CSF barrier is formed by the choroid plexus epithelium lining the floors of the 4 cerebral ventricles, and the 2 lateral ventricles are shown. The choroid plexus epithelium is a relatively leaky cellular barrier with a low electrical resistance of 26 Ω cm^2^. The CSF/plasma ratio of IgG is ∼0.2% (Table 1). Reproduced from Pardridge (2020).

It is widely believed that therapeutic antibodies have low, but significant, rate of penetration through the BBB, and that this brain uptake of the antibody produces a brain concentration on the order of 0.2% of the plasma concentration (Atwal et al, 2011; Bohrmann et al, 2012). In fact, this estimate is not based on measurement of antibody penetration of brain, but rather is derived from the observation that the level of therapeutic antibody in cerebrospinal fluid (CSF) is 0.2% of the plasma level following IV infusion. That is, the 0.2% estimate does not represent IgG uptake by brain, but rather IgG uptake by CSF. The use of drug distribution into CSF assumes the CSF compartment can be used as a surrogate of the brain interstitial fluid (ISF) compartment. This assumption on the equivalence of CSF and ISF in brain tissue overlooks important aspects of brain transport. CSF is not a surrogate of the ISF compartment, because the CSF is separated from blood by the choroid plexus epithelium, which forms the blood-CSF barrier. In contrast, ISF is separated from blood by the brain capillary endothelium of brain parenchyma, which forms the BBB. These 2 barriers in brain have fundamentally different properties as illustrated in Figure 2. The BBB is present at virtually all capillaries in the brain (Figure 2, left panel). Since the capillaries are separated by about 40 microns (Duvernoy et al, 1981), virtually all neurons are perfused by an individual capillary. In contrast, the blood-CSF barrier is localized only to the choroid plexus, which lines the floor of the 4 cerebral ventricles in brain. The choroid plexus at the floor of the 2 lateral ventricles is shown in Figure 2 (right panel). The BBB, at the brain capillary endothelium, and the blood-CSF barrier, at the choroid plexus, are distinct cellular barriers. The BBB is a tight barrier, with an electrical resistance of ∼8,000 Ω cm^2^, whereas the choroid plexus is a leaky barrier with an electrical resistance of 26 Ω cm^2^ (Pardridge, 2016). The relative leakiness of the choroid plexus, as compared to the BBB, is also reflected in the rate of transport into CSF of plasma proteins across the choroid plexus. As shown in Table 1, the CSF/serum ratio of albumin and IgG in humans is 0.58% and 0.25%, respectively (Kay et al, 1987). That is, *all* IgG in plasma normally transfers to the CSF compartment, owing to the relative leakiness of the choroid plexus. In contrast, the level of IgG in primate brain following IV administration is <0.01% of the plasma concentration (Yadav et al, 2017). The distribution of a therapeutic antibody into CSF is the expected consequence of the relatively leaky choroid plexus, and CSF measurements should not be used as an index of antibody transport across the BBB into brain parenchyma. The idea that CSF is a measure of brain ISF dates back to 1913, when the location of the BBB was erroneously assumed to be the choroid plexus (Pardridge, 2016).

**TABLE 1:** Concentration of IgG and albumin in human cerebrospinal fluid.

Compartment	IgG (mg/mL)	Albumin (mg/mL)
CSF	0.025 ± 0.010	0.22 ± 0.05
serum	9.8 ± 2.2	38 ± 4
CSF/serum	0.25%	0.58%

From Kay et al, 1987.

#### 2.2.2 Brain plasma volume

**FIGURE 3:**
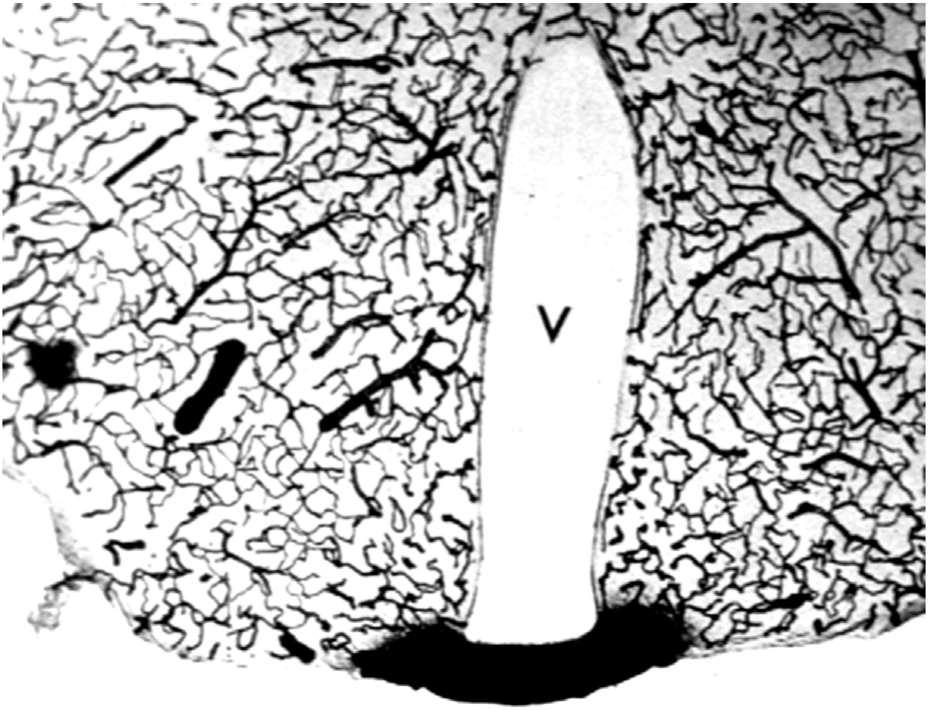
The brain plasma volume is illustrated in this coronal section of mouse brain after the IV injection of horseradish peroxidase (HRP), a 40 kDa protein that does not cross the BBB. Four tiny areas of brain, circumventricular organs (CVO), lack a BBB (Miyata, 2015), and one CVO, the median eminence at the base of the third ventricle (V), is shown in this section. The HRP is sequestered within the plasma compartment of brain without passage across the BBB. However, if a homogenate of brain was produced, then HRP in brain would be measurable. This image is a kind gift of Dr. Milton W. Brightman, and is reproduced from Pardridge (2022b).

Aducanumab was said to cross the intact BBB, because the brain concentration of the antibody was higher following the administration of a high injection dose (ID) as compared to the brain concentration of antibody following administration of a low ID (Sevigny et al, 2016). The aducanumab brain volume of distribution (VD), which is the ratio of the brain concentration, in ng/Gram, divided by the plasma concentration, in ng/uL, was only ∼1 uL/Gram, following washout of the brain (Sevigny et al, 2016). That is, the brain concentration of aducanumab was 1,000-fold lower than the plasma concentration. The brain VD of aducanumab, 1 uL/Gram, is 5% of the brain plasma volume in the mouse, which is ∼20 uL/g (Boswell et al, 2014). Therefore, this brain aducanumab concentration would be expected if 5% of the plasma volume was retained in brain following washout of the vasculature. If a therapeutic antibody, or any drug, is retained in brain within the plasma volume, then that drug is present in brain, *per se*, but has not crossed the BBB. This is illustrated by the histochemistry of mouse brain following the IV administration of horseradish peroxidase (HRP), as shown in Figure 3. HRP is a 40 kDa protein that does not cross the BBB, and does not enter brain, except in tiny regions of the brain, designated circumventricular organs (CVO), which lack a BBB (Miyata, 2015). One CVO is the median eminence at the base of the third ventricle, and the uptake of HRP in this region of brain is shown in Figure 3. If a homogenate of the brain shown in Figure 3 was prepared, then HRP would be measurable, and it might be concluded that HRP crosses the BBB, providing the role of the brain plasma volume was not considered. The role of the brain plasma volume (Vo) can generally be ignored in the development of small molecule drugs that have a VD in brain that is high relative to the Vo. However, in the case of drug development of biologics, where the brain VD approximates the Vo, it is crucial to account for drug sequestration in the plasma volume of brain, before concluding that a given biologic crosses the BBB. If the brain VD of a therapeutic antibody is not greater than the brain plasma volume, Vo, then the antibody does not cross the BBB.

#### 2.2.3 Brain microdialysis

Microdialysis of brain was originally developed to measure the distribution of small molecules into the ISF of brain, and employed the intra-cerebral implantation of a dialysis fiber with an outer diameter of ∼500 microns and a molecular weight cutoff (MWCO) of 20 kDa (Benveniste et al, 1989). Early work showed that the insertion of a dialysis fiber into brain causes local brain injury leading to BBB disruption to both small molecules (Morgan et al, 1996; Groothuis et al, 1998) and to large molecules such as albumin (Westergren et al, 1995), and to the formation of a glial scar around the fiber (Hascup et al, 2009). Recently, brain microdialysis has been applied to the brain delivery of monoclonal antibodies, where special fibers are used that have a MWCO of 1,000,000 Da (Chang et al, 2018; Sopko et al, 2020; LePrieult et al, 2021; Van De Vyver et al, 2022). The assumption with this methodology is that the antibody distribution into the dialysate is a measure of antibody transport into the brain ISF. However, the dialysate is only a measure of the ISF within a region of <1,000 microns surrounding the fiber (Mak et al, 1995). This peri-fiber region of brain is a model of a brain penetration injury, owing to the local stab wound caused by implantation of the fiber (Jaquins-Gerstl and Michael, 2009; Nesbitt et al, 2013; Varner, et al, 2016). The introduction of a fiber, which has an outer diameter of ∼500 microns (Chang et al, 2018), into brain tissue causes local ischemia around the fiber (Jaquins-Gerstl and Michael, 2009; Nesbitt et al, 2013; Varner, et al, 2016). The peri-fiber ischemia develops because the diameter of the fiber, ∼500 microns, is more than 10-fold greater than the inter-capillary distance, 40 microns, in brain (Duvernoy et al, 1981). The IV administration of 0.1 micron fluorescent microspheres in parallel with fiber implantation shows the brain capillaries adjacent to the dialysis fiber are not perfused (Jaquins-Gerstl and Michael, 2009). Owing to the BBB disruption caused by the implantation of the dialysis fiber within the region of brain surrounding the dialysis fiber, the use of this method will over-estimate the extent to which a therapeutic antibody crosses the BBB to enter brain parenchyma. Dialysate measurements represent antibody distribution in brain across a disrupted BBB within hypoxic, injured brain tissue.

In summary, therapeutic antibodies do not cross the intact BBB. Therefore, if the antibody target resides in the brain behind the BBB, the antibody must be enabled to penetrate the BBB. This is possible by re-engineering the therapeutic antibody as a BBB-penetrating bispecific antibody (BSA), where one antibody domain of the BSA is the therapeutic antibody and the other antibody domain of the BSA is a transporting antibody. The latter is an antibody that targets a receptor-mediated transcytosis (RMT) system at the BBB. These RMT pathways normally serve to mediate the brain uptake of specific peptides in blood. The BBB RMT systems may also mediate the brain uptake of receptor-specific MAbs that bind exofacial epitopes on the receptor extracellular domain. The RMT uptake of peptides, and receptor-specific MAbs, are reviewed in Sections 3.1 and 3.2, respectively. The re-engineering of therapeutic antibodies as BSAs that penetrate the BBB via these RMT pathways is reviewed in Section 3.3.

## 3 Receptor-mediated transport of biologics through the blood-brain barrier

### 3.1 Receptor-mediated transport of peptides through the blood-brain barrier

#### 3.1.1 Blood-brain barrier insulin receptor

The existence of receptor-mediated transcytosis (RMT) pathways at the BBB for circulating peptides was proposed following the identification of peptide RMT systems expressed in capillaries isolated from human and animal brain (Pardridge, 1986). The insulin receptor (INSR or IR) on the human BBB was identified with capillaries isolated from human autopsy brain, radio-receptor assays with [^125^I]-insulin, and affinity cross-linking of insulin to the IR at the human BBB (Pardridge et al, 1985). The high affinity KD of insulin binding to the BBB human insulin receptor (HIR) was 1.2 ± 0.5 nM. Affinity cross-linking studies showed the molecular weight (MW) of the insulin binding site was 127 kDa, and the solubilized BBB HIR bound to wheat germ agglutinin (WGA) indicating the presence of N-acetylglucosamine residues within the receptor carbohydrate moiety. In contrast, the HIR on brain cell membranes, depleted of brain capillaries, had a lower MW of 115 kDa, and did not bind to WGA (Pardridge et al, 1985). There are 2 HIRs, derived from a common gene. The short form receptor, isoform A, or HIR-A, lacks the domain expressed by exon 11, and is primarily expressed in neurons, whereas the long form receptor, isoform B, or HIR-B, contains the domain expressed by exon 11, and is primarily expressed in peripheral tissues and liver (Belfiore et al, 2009; Pomytkin et al, 2018). The HIR isoform expressed at the endothelium of brain may be the short form, or HIR-A. HIR-A has high affinity for insulin-like growth factor (IGF)-2, but not for IGF-1, whereas HIR-B has low affinity for IGF2 (Belfiore et al, 2017). IGF-2 partially suppresses insulin binding to the HIR at the human BBB (Duffy et al, 1988), which suggests the IR at the human BBB may be HIR-A.

The HIR at the human brain capillary mediates insulin endocytosis and exocytosis (Pardridge et al, 1985), and these observations gave rise to the hypothesis that insulin undergoes receptor-mediated transcytosis (RMT) across the BBB following binding to the luminal IR (Pardridge, 1986). This hypothesis was confirmed by internal carotid artery infusion of [^125^I]-insulin followed by emulsion autoradiography of brain in the rabbit (Duffy and Pardridge, 1987). Reverse phase HPLC of acid ethanol extracts of brain showed the radioactive species in brain tissue following carotid artery infusion was unmetabolized insulin. The brain uptake of the [^125^I]-insulin was completely saturated by high concentrations of unlabeled insulin (Duffy and Pardridge, 1987). The only binding site for insulin at the BBB that is saturable is the IR (Pardridge et al, 1985), indicating the IR on the BBB mediates the brain uptake of insulin. The transport of [^125^I]-insulin across the BBB *in vivo* was assessed by internal carotid artery infusion, and not by intravenous injection, because the plasma T_1/2_ of insulin is only about 5 min (Duckworth et al, 1998). This rapid peripheral degradation of [^125^I]-insulin results in the entry of [^125^I]-tyrosine into the circulation, since iodination labels tyrosine residues. Tyrosine is a large neutral amino acid that can traverse the BBB via transport on the large neutral amino acid transporter 1 (LAT1) that is highly expressed at the BBB (Boado et al, 1999). The IV injection of [^125^I]-insulin can lead to radioactivity in brain that arises from the BBB transport of [^125^I]-tyrosine, in parallel with the transport of [^125^I]-insulin.

#### 3.1.2 Blood-brain barrier transferrin receptor

High expression of the transferrin receptor (TfR) at the brain capillary endothelium was demonstrated by immunohistochemistry (IHC) of rat brain with the murine OX26 MAb against the rat TfR, and of human brain with the murine B3/25 MAb against the human TfR (Jefferies et al, 1984). Binding of the B3/25 antibody to the human TfR was originally described by Trowbridge and Omary (1981). Transferrin binding and endocytosis via the TfR at the human BBB was identified with isolated human brain capillaries and radio-receptor assays with [^125^I]-holo-Tf (Pardridge et al, 1987a). Holo-Tf bound to the human brain capillary with high affinity and a KD of 5.6 ± 1.4 nM, and the [^125^I]-holo-Tf was endocytosed into the brain capillaries via a temperature-dependent process. The expression of the TfR in the isolated human brain capillaries was confirmed with immunocytochemistry using a mouse MAb against the human TfR (Pardridge et al, 1987a). The RMT of [^125^I]-holo-Tf across the BBB in the rat was demonstrated with an arterial infusion method (Fishman et al, 1987). Subsequently, the brain uptake of [^125^I, ^59^Fe]-holo-Tf was measured in rats for up to 6 h after IV injection, and there was selective enrichment in brain of the ^59^Fe relative to the ^125^I (Taylor et al, 1991). These data, in conjunction with pre-embedding electron microscopic immunochemistry of brain showing absence of the TfR on the abluminal membrane of the capillary endothelium (Roberts et al, 1993), gave rise to the retro-endocytosis model of BBB transport of holo-Tf. In this model, it was posited that holo-Tf undergoes receptor-mediated endocytosis at the luminal membrane, followed by separation of iron and apo-Tf within the intracellular compartment of the endothelium, followed by retro-endocytosis of apo-Tf across the luminal endothelial membrane back to blood. However, the detection of abluminal receptors on the brain endothelium is limited with the pre-embedding labeling methods used by Roberts et al (1993), as abluminal receptors at the brain endothelium are best detected with post-embedding labeling methods (Vorbrodt, 1989). The expression of the TfR on the abluminal membrane of the endothelium was confirmed with confocal microscopy of freshly isolated rat brain capillaries (Huwyler and Pardridge, 1998). The selective enrichment of ^59^Fe, relative to ^125^I, at 6 h following IV injection (Taylor et al, 1991), is compatible with a model of RMT of holo-Tf across the BBB, uptake of the holo-Tf by brain cells, dissociation of iron in brain cells, and reverse transcytosis of apo-Tf from brain back to blood across the BBB. The selective reverse transcytosis of apo-Tf from brain to blood was demonstrated with the Brain Efflux Index method, and these studies showed the T_1/2_ of apo-Tf exodus from brain to blood was 49 ± 4 min (Zhang and Pardridge, 2001).

There are 2 TfRs, TfR1 and TfR2 (Kawabata et al, 1999). TfR1 is enriched in spleen relative to liver, and TfR2 is enriched in liver relative to spleen (Wallace et al, 2005). TfR1 is encoded by a 5–6 kb mRNA, and TfR2 is encoded by a 2.8 kb mRNA (Kawabata et al, 1999). The TfR expressed at the BBB is TfR1, as demonstrated by an early BBB genomics study (Li et al, 2001). The plasma concentration of Tf is very high, 45,000 nM (Schmaier, 2020), and about 40% of the total Tf in plasma is apo-Tf, about 30% is diferric holo-Tf, and about 30% is monoferric holo-Tf (Eckenroth et al, 2011). Apo-Tf does not bind the TfR at neutral pH, and the affinity of diferric holo-Tf for the TfR is about 6-fold greater than the affinity of mono-ferric holo-Tf (Mason et al, 2009). The complex of holo-Tf and the TfR is a tetramer comprised of 2 TfRs and 2 holo-Tfs (Eckenroth et al, 2011). Holo-Tf binds to the protease-like and helical domains of the TfR (Eckenroth et al, 2011). Although holo-Tf does not bind the apical domain of the TfR, the binding of holo-Tf to the protease-like and helical domains causes a conformational change in the apical domain of the TfR (Eckenroth et al, 2011; Kleven et al, 2018). The conformational change in the apical domain caused by Tf binding to the TfR is important with respect to TfRMAb binding to the TfR, as these antibodies bind the apical domain of the TfR, as discussed in section 3.2.3.

#### 3.1.3 Blood-brain barrier insulin-like growth factor receptor

The insulin-like growth factor (IGF) receptor at the human BBB was characterized with isolated human brain capillaries using radio-receptor assays with [^125^I]-IGF1 and [^125^I]-IGF2 and affinity cross-linking of the IGFs to the human brain capillary plasma membrane (Duffy et al, 1988). Binding of the IGFs to the human BBB was high affinity and the KD of binding of IGF1 and IGF2 was 2.1 ± 0.4 nM and 1.1 ± 0.1 nM, respectively. Subsequent to receptor binding, the IGFs were rapidly endocytosed into the human brain capillary. IGF1 binds with high affinity to the IGF1 receptor (IGF1R), but does not bind to the insulin receptor or to the IGF2 receptor (IGF2R). The IGF2R is also known as the cation independent mannose 6-phosphate receptor (CI M6PR), and binds mannose 6-phosphorylated lysosomal enzymes (Pardridge, 2022a). IGF2 binds with high affinity to the IGF1R, the IGF2R, and the type A (short form) insulin receptor (Jiracek and Zakova, 2017). The IGF1R and insulin receptors are hetero-tetameric tyrosine kinases, whereas the IGF2R is a monomeric 250 kDa protein (Van Beijnum et al, 2017). Both IGF1 and IGF2 bind to a saturable binding site at the human BBB that has a MW of 140 kDa, which is size of the alpha subunit of the IGF1R, and there is no detectable 250 kDa binding site at the human BBB for IGF2 (Duffy et al, 1988). The absence of the IGFR2/CI M6PR at the human BBB underlies the lack of BBB transport of lysosomal enzymes, which are mannose 6-phosphorylated (Pardridge, 2022a).

The BBB IGF1R mediates the saturable transport of both IGF1 and IGF2 across the BBB *in vivo*, as demonstrated with an internal carotid artery infusion in rats of [^125^I]-IGF1 and [^125^I]-IGF2 in buffer without serum (Reinhardt and Bondy, 1994). The brain uptake of [^125^I]-IGF1 or [^125^I]-IGF2 would not be measurable following IV administration, because the IGFs are >99% bound by IGF binding proteins (IGF BP) (Clemmons, 2018). IGF1 and IGF2 binding to the IGFBPs sequester the IGFs in the blood compartment, which explains the long T_1/2_ of the IGFs in blood of 10–16 h (Livingstone and Borai, 2014). In contrast, the T_1/2_ of insulin in blood is 4–5 min (Duckworth et al, 1998). There is no specific binding of either IGF1 or IGF2 to IGF1R on isolated human brain capillaries in the presence of human serum (Duffy et al, 1988). The avid binding of the IGFs to serum IGFBPs explains the lack of BBB transport of an IGF2 fusion protein (Kan et al, 2014). IGF2 is synthesized locally at the brain microvasculature as demonstrated by a BBB genomics study of isolated rat brain capillaries (Li et al, 2001).

#### 3.1.4 Blood-brain barrier leptin receptor

**FIGURE 4:**
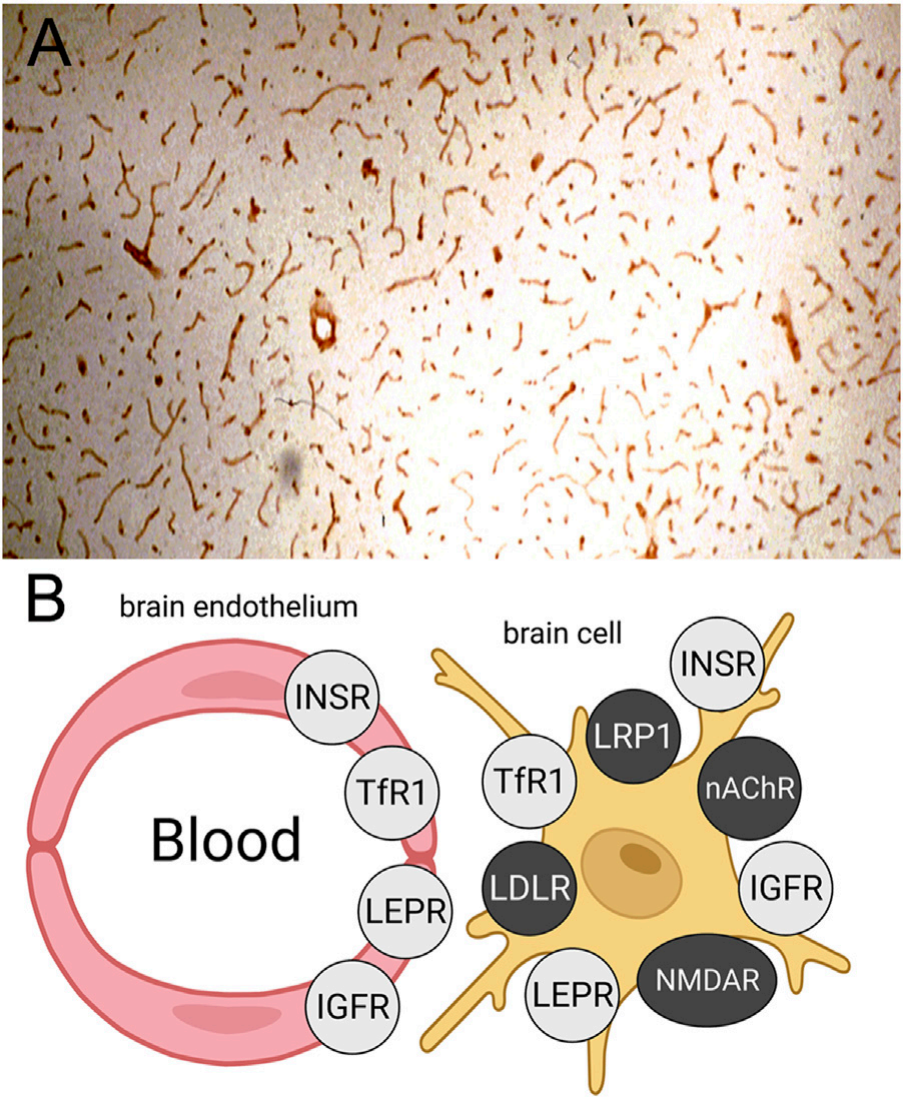
**(A)** Immunohistochemistry (IHC) of rat brain with an antiserum against the leptin receptor (LEPR) shows high expression of the LEPR at the brain microvasculature. The continuous immune staining is indicative of an endothelial origin of the immunoreactive LEPR. Reproduced with permission from Boado et al (1998). **(B)** Schematic showing expression of the insulin receptor (INSR), the transferrin receptor 1 (TfR1), the LEPR, and the insulin-like growth factor receptor (IGFR) at both the BBB and on brain cell membranes. Conversely, brain IHC shows the low density lipoprotein (LDL) receptor (LDLR), the LDLR related protein 1 (LRP1), the nicotinic acetyl choline receptor (nAChR), and the N-methyl D-aspartate receptor (NMDAR) are expressed on brain cells but not on the luminal membrane of the brain capillary endothelium. Reproduced from Pardridge (2022a).

The leptin receptor (LEPR), also called the obesity receptor (Ob-R), was identified at the human BBB with isolated human brain capillaries and radio-receptor assays with [^125^I]-leptin (Golden et al, 1997). The KD of binding of leptin to the BBB LEPR was 5.1 ± 2.8 nM, and leptin binding was not inhibited by either insulin or IGF1 (Golden et al, 1997). Reverse transcriptase polymerase chain reaction (PCR) with RNA freshly isolated from rat brain capillaries showed the predominant Ob-R at the BBB was the short form of the receptor, Ob-Ra (Boado et al, 1998), which has a truncated intracellular domain (Gorska et al, 2010). The selective expression of the LEPR at the BBB in brain is shown by the IHC study in Figure 4A, where frozen sections of rat brain were immune-stained with an antibody against the LEPR (Boado et al, 1998). The continuous immune-staining of the brain microvasculature is indicative of an endothelial origin of the LEPR. In contrast a discontinuous immune staining of a capillary antigen generally indicates the cellular origin of the antigen is either the microvascular pericyte or microvascular astrocytic endfeet.

The BBB LEPR mediates the brain uptake of circulating leptin as demonstrated with carotid artery infusion of [^125^I]-leptin (Kurrimbux et al, 2004). Leptin penetrated the parenchyma of brain faster than the peptide entered into CSF (Kurrimbux et al, 2004). BBB transport of leptin was suppressed following selective knockout of the brain capillary endothelial LEPR (Di Spezio et al, 2018). The demonstration of receptor-mediated transport of [^125^I]-leptin following IV administration is made difficult owing to the short half-time, 40 min, of leptin in plasma (Burnett et al, 2017). Degradation of [^125^I]-leptin results in the rapid formation of [^125^I]-tyrosine in plasma, and this large neutral amino acid is a substrate for the BBB LAT1 transporter (Boado et al, 1999). BBB transport of leptin has been assessed by fluorescent microscopy following the IV injection of leptin conjugated with a fluorophore (Harrison et al, 2019). The delivery of leptin to brain was detected in regions with a leaky vasculature, such as the choroid plexus or a CVO (Harrison et al, 2019). Transfer of leptin from blood to brain parenchyma behind the BBB with a microscopic method is made difficult by the marked dilution the peptide undergoes in transfer from the intra-endothelial volume of brain to the post-vascular volume of brain. The post-vascular space of brain, 700 uL/Gram, is ∼1,000-fold larger than the intra-endothelial volume, 0.8 uL/Gram (Pardridge, 2022b).

#### 3.1.5 Other receptors

Immunohistochemistry (IHC) of brain shows the insulin, transferrin, IGF, and leptin receptors are expressed both at the BBB and on cells within brain parenchyma, as depicted in Figure 4B. IHC of brain with receptor-specific antibodies shows continuous immune staining of the brain with antibodies against the IR (Pardridge et al, 1995; Kurata et al, 2013), the TfR (Jefferies et al, 1984; Bickel et al, 1994; Huwyler and Pardridge, 1998), the IGF1R (Garcia-Segura, 1997), and the LEPR (Boado et al, 1998). In contrast, IHC of brain shows several receptors, which have been targeted for RMT across the BBB with peptide-based delivery agents, are expressed on brain cells, but not at the endothelium, as shown in Figure 4B. The low-density lipoprotein (LDL) receptor (LDLR) is expressed on neurons but not endothelium (Kurata et al, 2013). The LDLR related protein 1 (LRP1) is expressed on pericytes (Candela et al, 2015; Ma et al, 2018) and astrocytes (Liu et al, 2017) but not endothelium of brain. The nicotinic acetyl choline receptor (nAChR) is expressed on neurons and astrocytes in brain but not on endothelium (Gahring et al, 2004). The N-methyl D-aspartate receptor (NMDAR) is expressed on pericytes but not endothelium (Mapelli et al, 2017). IHC of brain shows the IR, TfR, LEPR, and IGF1R are also expressed within brain cells. The IR is expressed on neurons and astrocytes (Pomytkin et al, 2018), the TfR is expressed on neurons (Moos et al, 1998), the LEPR is expressed on neurons and astrocytes (Mutze et al, 2006; Fujita and Yamashita, 2019), and the IGF1R is expressed on neurons and astrocytes (Garcia-Segura, 1997). Therefore, targeting the IR, TfR, LEPR, or IGF1R could deliver therapeutic antibodies to the intracellular compartment of brain. Pathologic aggregates accumulate in the intracellular compartment of brain, such as neurofibrillary tangles in AD (Kuchibhotla et al, 2014) or α-synuclein aggregates in PD (Peelaerts and Baekelandt, 2023). In contrast, the development of antibodies that target the LDLR, the LRP1, the nAChR, or the NMDAR, may not result in significant uptake of the antibody by brain, as these receptors are not expressed on the luminal membrane of the brain capillary endothelium. An antibody specific for the LRP1 is not transported into brain following IV administration (Zuchero et al, 2016). Other receptors at the BBB that have been proposed as potential targets for RMT delivery include basigin (Christensen et al, 2021) and CD98hc (Zuchero et al, 2016). However, both basigin and CD98hc are not RMT systems, but rather are members of the Solute Carrier (SLC) gene family of carrier-mediated transporters (Schumann et al, 2020). Basigin, also known as CD147, forms a hetero-dimer with the monocarboxylic acid transporter, MCT1 (SLC16A1) (Wilson et al, 2002). CD98hc, also known as 4F2hc, is SLC3A2 and forms a hetero-dimer with the large neutral amino acid transporter 1, LAT1 (SLC7A5). The LAT1/4F2hc hetero-dimer, and the MCT1/CD147 hetero-dimer, form trans-membrane transport cavities (Yan et al, 2019; Wang et al, 2021) and do not undergo endocytosis (Pardridge, 2022b).

#### 3.1.6 Differential distribution of insulin and transferrin receptors at the blood-brain barrier

The local capillary concentration of the IR and TfR at the BBB is 24 nM and 40 nM, respectively (Pardridge, 2021). The plasma concentration of insulin, 0.3 nM (Bar et al, 1976), is only 1% of the capillary IR concentration. Therefore, over 80% of the IR at the BBB is the free IR not occupied by insulin (Pardridge, 2021). In contrast, the concentration of holo-Tf in plasma, 25,000 nM (Schmaier, 2020), is nearly 1,000-fold greater than the concentration of the TfR at the BBB (Pardridge, 2021). Therefore, the concentration of the free TfR, unoccupied by Tf, at the BBB is effectively zero, and all of the TfR at the BBB is in the form of a hetero-tetrameric complex of 2 Tf molecules and 2 receptors. Most of the endothelial TfR resides within the intracellular compartment participating in the transcytosis of Tf, and the concentration of the TfR-Tf complex at the luminal membrane of the brain endothelium is estimated to be 2 nM (Pardridge, 2021). The fact that a TfRMAb antibody targets the TfR-Tf complex *in vivo* at the BBB, and not the unoccupied TfR, is important to TfRMAb discovery. This is because binding of holo-Tf to the TfR causes conformational changes in the apical domain of the TfR (Eckenroth et al, 2011; Kleven et al, 2018), and it is the apical domain of the TfR where virtually all TfRMAbs bind. Therefore, the affinity of a TfRMAb for the TfR should be assessed for both the unoccupied TfR and the holo-Tf/TfR complex. Since the affinity for the TfR of diferric Tf is about 6-fold greater than monoferric Tf (Mason et al, 2009), it is important to use a complex of the TfR and diferric Tf when measuring the affinity of a TfRMAb for the TfR.

### 3.2 Receptor-mediated transport of monoclonal antibodies through the blood-brain barrier

#### 3.2.1 Insulin receptor monoclonal antibodies

A murine MAb that binds the extracellular domain (ECD) of the alpha subunit of the human insulin receptor (HIR), and designated the HIRMAb, was observed to bind with high affinity to isolated human brain capillaries with a KD of 0.45 ± 0.10 nM (Pardridge et al, 1995). HIRMAb binding to human brain capillaries was minimally affected by insulin and the concentration of insulin that caused a 50% inhibition of HIRMAb binding was >2,000 nM. Subsequent to binding, the HIRMAb was rapidly endocytosed by the human brain capillary. IHC with primate brain showed the HIRMAb cross-reacted with the IR in Old World primates, such as the Rhesus monkey, but not the IR in New World primates, such as the squirrel monkey. The HIRMAb was radio-iodinated and injected IV in the adult Rhesus monkey. The brain uptake of the HIRMAb by the primate brain was 2.5–3.8%ID/100 g (Pardridge et al, 1995). Brain uptake was expressed per 100 g, because the weight of the primate brain is 100 g (Pardridge et al, 1995). This level of brain uptake of the HIRMAb in the primate is high and is comparable to the brain uptake of a lipid soluble small molecule. Fallypride is a lipid soluble small molecule dopamine receptor agonist, and the brain uptake of fallypride in the primate is 4%ID/100 g (Mukherjee et al, 2001). The murine HIRMAb was genetically engineered as a human/mouse chimeric antibody (Coloma et al, 2000). The chimeric HIRMAb was radio-labeled by conjugation with diethylenetriaminepentaacetic acid (DTPA) followed by chelation of the 111-indium radionuclide. The brain uptake of the [^111^In-DTPA]-chimeric HIRMAb in the Rhesus monkey was 2.0 ± 0.1 %ID/100 g at 2 h after IV administration. Film autoradiography of the primate brain removed 2 h after injection showed global distribution of the chimeric HIRMAb throughout the brain with higher uptake in gray matter as compared to white matter (Coloma et al, 2000), owing to the higher vascular density in gray matter (Pardridge et al, 1995).

**FIGURE 5:**
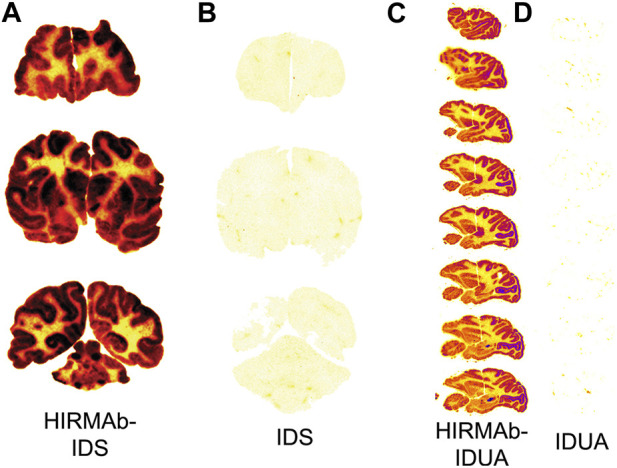
**(A, B)** Film autoradiography of coronal sections of Rhesus monkey brain removed 2 h after the IV injection of [^125^I]-HIRMAb-IDS fusion protein **(A)** or [^125^I]-IDS **(B)**. Reproduced with permission from Boado et al (2013a) **(C, D)** Phosphorimager scans of sagittal sections of Rhesus monkey brain removed 2 h after the IV injection of [^125^I]-HIRMAb-IDUA fusion protein **(C)** or [^125^I]-IDUA **(D)**. Reproduced with permission from Boado and Pardridge (2017). Fusion of the IDUA or IDS enzyme to the HIRMAb enables broad penetration of the enzyme into the brain parenchyma. Uptake in gray matter is higher than in white matter owing to the greater vascular density in gray matter. The IDS **(B)** or IDUA **(D)** alone does not cross the BBB and is confined to the plasma volume of brain.

A fusion protein of the chimeric HIRMAb was genetically engineered where the mature iduronate 2-sulfatase (IDS) enzyme was fused to the carboxyl terminus of both heavy chains. IDS is a lysosomal enzyme mutated in Mucopolysaccharidosis Type II (MPSII), which affects the CNS (Pardridge, 2022a). The brain uptake of the IDS alone or the HIRMAb-IDS fusion protein was measured in Rhesus monkeys at 2 h after IV administration of the enzyme or fusion protein radio-labeled with the [^125^I]-Bolton-Hunter reagent. The brain uptake of the IDS and HIRMAb-IDS fusion protein was 0.026 ± 0.007%ID/100 g and 1.0% ± 0.2% ID/100 g (Boado et al, 2013a), respectively. The brain VD of the IDS was 9 ± 2 uL/Gram, which is no different from the plasma volume. Therefore, the IDS enzyme does not cross the BBB, and the brain uptake of IDS, 0.026 ± 0.007 %ID/100 g, represents enzyme entrapment in the brain plasma volume (Boado et al, 2013a). Film autoradiography of the primate brain at 2 h after IV injection is shown in Figure 5A and 5B for the HIRMAb-IDS fusion protein and IDS, respectively (Boado et al, 2013a). Similar to the global uptake by primate brain of the murine or chimeric HIRMAb alone (Pardridge, et al, 1995; Coloma et al, 2000), there is broad distribution in primate brain of the HIRMAb-IDS fusion protein (Figure 5A). Conversely, there is no brain uptake of the IDS alone (Figure 5B). In a parallel study, the lysosomal enzyme mutated in MPSI, α-L-iduronidase (IDUA), was fused to the carboxyl terminus of both heavy chains of the chimeric HIRMAb (Boado and Pardridge, 2017). The IDUA alone and the HIRMAb-IDUA fusion protein were separately labeled with the [^125^I]-Bolton-Hunter reagent, and injected IV in the Rhesus monkey. The brain VD of the HIRMAb-IDUA fusion protein was 736 ± 59 uL/Gram, which corresponded to a brain uptake of 1.2% ± 0.2% ID/100 g. Conversely, the brain VD of the IDUA alone, 14 ± 1 uL/Gram, was no different from the brain plasma volume (Boado and Pardridge, 2017). Phosphor imaging of the primate brain at 2 h after IV injection is shown in Figures 5C, D for the HIRMAb-IDUA fusion protein and IDUA, respectively (Boado et al, 2013a). Similar with the HIRMAb-IDS fusion protein, there is global uptake by brain of the HIRMAb-IDUA fusion protein, with higher uptake in gray matter as compared to white matter. The images in Figure 5 illustrate the extent to which a biologic, which alone does not cross the BBB, can be re-engineered with a HIRMAb to enable brain uptake of the biologic.

The robust brain uptake of the HIRMAb-enzyme fusion proteins shown in Figures 5A, C would not be observed if the high affinity, bivalent HIRMAb was sequestered within the brain capillary endothelium without exocytosis into the brain parenchyma. If this were the case, then the brain uptake of the HIRMAb fusion protein would be at the background level observed for the IDS and IDUA alone (Pardridge, 2021). This is because the volume of the capillary endothelium in brain, 0.8 uL/Gram (Gjedde and Christensen, 1984), is nearly 1,000-fold smaller than the extravascular volume of brain, 700 uL/Gram (Pardridge, 2022b). Owing to this tiny volume of the intracellular space of the capillary endothelium in brain, the sequestration of the HIRMAb within the endothelium, without exocytosis into post-vascular brain, would produce only a background level of brain uptake (Pardridge, 2021). The rapid transcytosis of HIRMAb fusion proteins across the primate BBB was confirmed with the capillary depletion method (Boado et al, 2013a; Boado and Pardridge, 2017), and by light microscopic emulsion autoradiography of primate brain (Boado et al, 2013b).

The safety pharmacology and toxicology of HIRMAb fusion proteins was tested in over 70 Rhesus monkeys following chronic IV administration at doses up to 30 mg/kg weekly for 6 months (Pardridge, 2022a). The only adverse event was hypoglycemia following rapid IV infusion of a high dose, 30 mg/kg, of the HIRMAb-IDUA fusion protein in saline without dextrose, and this hypoglycemia was eliminated by addition of dextrose to the infusate (Pardridge, 2022a). Chronic treatment of primates for 6 months with 30 mg/kg weekly caused no impairment in glycemic control (Pardridge, 2022a). A detailed neuropathologic exam of the brains of all monkeys showed no neurodegeneration, based on hematoxylin and eosin staining or fluoro-Jade B fluorescent microscopy, and no astrogliosis based on glial fibrillary acidic protein (GFAP) IHC (Pardridge, 2022a) The first clinical trial with a BBB Trojan horse antibody was reported by Giugliani et al (2018). The HIRMAb-IDUA fusion protein, designated valanafusp alfa, was infused primarily at doses of 1–3 mg/kg weekly for 1 year in 11 pediatric subjects with MPSI. Treatment stabilized the decline in Developmental Quotient (DQ) and brain atrophy associated with CNS involvement in MPSI. The incidence of mild infusion related reactions or hypoglycemia was <2% over the course of 52 weeks of treatment (Giugliani et al, 2018). The HIRMAb was used to produce the first BBB-penetrating BSA of a therapeutic antibody as described in Section 3.3.1.

#### 3.2.2 IGF1 receptor monoclonal antibodies

A IGF1RMAb was isolated as a camelid VHH nanobody following immunization of a llama with recombinant human IGF1R ECD (Yogi et al, 2022). The camelid VHH bound to the human IGF1R with high affinity, KD = 0.4–1.1 nM, across species. However, there was, on average, an 18-fold reduction in affinity of the VHH following humanization of the nanobody framework regions. The non-humanized camelid VHH, which has a MW of 15 kDa, was fused to the carboxyl terminus of both chains of human Fc to produce an 80 kDa Fc-VHH fusion protein, which was injected IV in rats at a dose of 15 mg/kg. At 24 h after injection, the CSF/serum ratio of the antibody was 0.3% and the brain antibody concentration was 11 nM (Yogi et al, 2022). Based on the injection dose, the brain concentration of 11 nM corresponds to a brain uptake of 0.04%ID/Gram, which suggests the IGF1R at the BBB was saturated by the high ID of 15 mg/kg of the high affinity Fc-VHH fusion protein.

A single chain (scFv) antibody against the human IGF1R was isolated following screening of a human scFv library with human IGF1R ECD (Shin et al, 2022). Affinity maturation of the scFv produced an antibody, designated Grabody B, with a KD of binding to the human IGF1R of 1–6 nM across several species, as determined by surface plasmon resonance. The concentration of antibody that produced 50% binding to the IGF1R, or EC50, as determined by ELISA, was 0.13–0.4 nM for the Grabody B (Shin et al, 2022). The Grabody B was then used to produce a BSA that targets the IGF1R on the BBB, and α-synuclein as the therapeutic antibody, as discussed in Section 3.3.4.

#### 3.2.3 Transferrin receptor monoclonal antibodies

The murine OX26 MAb against the rat TfR binds the brain capillary endothelium as shown by IHC of rat brain (Jefferies et al, 1984). The extent to which the OX26 antibody crosses the BBB *in vivo* was tested in rats following tritiation of the antibody with [^3^H]-sodium borohydride (Pardridge et al, 1991). In parallel, the isotype control antibody, mouse IgG2a, was radio-iodinated. The brain VD of the mouse IgG2a was 11 ± 1 uL/Gram, which indicates this control IgG is confined to the brain plasma volume. In contrast, the brain VD of the OX26 TfRMAb was 200 ± 10 uL/Gram, and capillary depletion studies showed the OX26 antibody transcytosed across the rat BBB *in vivo* (Pardridge et al, 1991). The neuropharmacologic activity of the OX26 antibody as a BBB Trojan horse delivery agent was tested with *in vivo* cerebral blood flow (CBF) assays in rats (Bickel et al, 1993). Vasoactive intestinal peptide (VIP) is a potent vaso-dilator of brain vessels following topical administration. However, VIP does not cross the BBB, and the intra-carotid arterial infusion of VIP caused no change in CBF, although the arterial infusion of the peptide increased thyroid blood flow by 89% (Bickel et al, 1993). When the VIP was conjugated to the OX26 antibody via an avidin-biotin linker, a 65% increase in CBF was observed following infusion of the VIP-OX26 conjugate (Bickel et al, 1993). The ID of the VIP was 12 ug/kg and the ID of the OX26-avidin conjugate was 0.43 mg/kg (Bickel et al, 1993). These studies showed that high affinity, bivalent TfRMAbs, such as OX26, could induce *in vivo* pharmacologic effects in the brain following the RMT of the antibody-drug complex across the BBB. In another early pharmacologic study, nerve growth factor was chemically conjugated to the OX26 antibody and this conjugate was neuroprotective in an intra-ocular neural transplant model in the rat (Friden et al, 1993). The RMT of a conjugate of the OX26 MAb and 5 nm gold was assessed by electron microscopy, which showed the antibody was delivered through the endothelium within 50–100 nm vesicles, followed by exocytosis into brain ISF (Bickel et al, 1994). The rapid RMT of either holo-Tf or the OX26 antibody across the BBB in the rat was demonstrated with internal carotid artery infusion followed by capillary depletion or light microscopic emulsion autoradiography (Skarlatos et al, 1995). Both holo-Tf and the OX26 antibody rapidly entered brain ISF within just 5 min of carotid arterial infusion at comparable rates of transcytosis (Skarlatos et al, 1995). The rapid RMT of either holo-Tf or the OX26 antibody across the BBB is due, in part, to the minimal thickness of the brain capillary endothelium, which is only 300 nm thick (Cornford et al, 1998). In contrast, the thickness of the choroid plexus epithelium, 10 microns (Liddelow et al, 2010), is over 30-fold greater than the thickness of the BBB. The genes encoding the variable region of the heavy chain (VH) and variable region of the light chain (VL) of the OX26 MAb were cloned and sequenced to produce the amino acid sequences of the VH and VL of the OX26 antibody (Li et al, 1999).

The OX26 antibody is active only in the rat, and does not recognize the mouse TfR (Lee et al, 2000). The 8D3 or RI7-217 rat antibodies against the mouse TfR rapidly enter mouse brain after IV injection. The mouse brain uptake of the 8D3 and RI7-217 antibodies at 60 min after administration was 3.1 ± 0.4 %ID/Gram and 1.6 ± 0.2 %ID/Gram (Lee et al, 2000), respectively. In contrast, the distribution of the OX26 antibody in mouse brain was confined to the plasma volume, and the brain uptake was only 0.06 ± 0.01 %ID/Gram. The brain uptake of the high affinity 8D3 TfRMAb in the mouse was nearly 90% saturated by an intravenous ID of 4 mg/kg (Lee et al, 2000). Following the determination of the amino acid sequence of the heavy and light chains of the 8D3 antibody, the rat VH and VL were grafted on to constant domains of mouse IgG1 heavy chain and mouse kappa light chain to produce a rat/mouse chimeric form of the 8D3 antibody (Boado et al, 2009). The high affinity binding of the chimeric 8D3 antibody to the mouse TfR was retained following the genetic engineering as the EC50 of binding of the 8D3 antibody and the chimeric antibody was 2.3 ± 0.3 nM and 2.6 ± 0.3 nM, respectively (Boado et al, 2009). The brain uptake of the chimeric TfRMAb is comparable to the brain uptake of the 8D3 antibody in the mouse, and both antibodies have a brain uptake of 2%–3% ID/Gram (Boado et al, 2010). This level of brain uptake of the TfRMAb in the mouse is high and approximates the brain uptake of a lipid soluble small molecule, diazepam, which has a brain uptake in the mouse of ∼5% ID/Gram (Greenblatt and Sethy, 1990).

A variable domain of the new antigen receptor (VNAR) single domain antibody against the TfR1, designated TXB2, was isolated following the screening of a phage library of nurse shark VNAR with human TfR1 ECD (Stocki et al, 2021). The VNAR antibody was fused to the amino terminus of human Fc to produce a bivalent VNAR-derived TfRMAb. The VNAR-hFc bound with high affinity to human, cynomolgus monkey, mouse, and rat TfR1, as determined by surface plasmon resonance, and the KD ranged from 0.28 nM, for the monkey, to 1.2 nM for the rat. The brain uptake in the mouse was high and peaked at 6 nM following the IV injection of 1.9 mg/kg, which is equivalent to a brain uptake of 0.65 %ID/Gram, and transcytosis of the high affinity, bivalent TfRMAb across the BBB was confirmed with the capillary depletion method (Stocki et al, 2021).

#### 3.2.4 Affinity and valency of transferrin receptor monoclonal antibodies

The affinity of genetically engineered TfRMAbs, which have been developed by multiple laboratories as BBB Trojan horse delivery agents, varies over 4 log orders of magnitude. High affinity TfRMAbs have a binding KD of 0.1–10 nM (Boado et al, 2009; Pardridge et al, 2018; Sonoda et al, 2018; Do et al, 2020; Stocki et al, 2021), moderate affinity TfRMAbs have a binding KD of 10–50 nM (Niewoehner et al, 2014; Yu et al, 2014; Do et al, 2020), low affinity TfRMAbs have a binding KD of 50–1,000 nM (Yu et al, 2011; Kariolis et al, 2020; Ullman et al, 2020), and very low affinity TfRMAbs have a binding KD of 1,000–2,000 nM (Kariolis et al, 2020; Van Lengerich et al, 2023). Yu et al (2011) hypothesized that a low affinity TfRMAb was a preferred BBB delivery agent, relative to a high affinity TfRMAb, because the high affinity TfRMAb was said to be sequestered within the brain endothelium, following endocytosis from blood. This hypothetical sequestration was said to cause minimal exocytosis of the TfRMAb from the endothelium to brain ISF. This hypothesis was based on two lines of evidence. First, the brain uptake of a low affinity TfRMAb was greater than the brain uptake of a high affinity TfRMAb following the administration of a very high ID of 30–50 mg/kg (Yu et al, 2011). This high ID, 30–50 mg/kg, was considered a ‘therapeutic dose,’ whereas an ID of 4 mg/kg was considered a ‘low dose.’ However, the higher brain uptake of a low affinity TfRMAb at an ID of 30–50 mg/kg is the expected result. It was shown over 10 years earlier that the brain uptake of a high affinity TfRMAb was saturated by an ID of 4 mg/kg (Lee et al, 2000). Moreover, a therapeutic dose of a high affinity, bivalent TfRMAb is only 1 mg/kg, as discussed below. Therefore, an ID of 30–50 mg/kg, which is 10-fold higher than the ID that fully saturates brain uptake of a high affinity TfRMAb, is not expected to produce higher brain uptake for the high affinity TfRMAb, whereas the brain uptake of a low affinity TfRMAb is not saturated by the high ID of 30–50 mg/kg. Second, IHC of brain detected the TfRMAb only in the brain endothelium, and not in the post-vascular compartment of brain, a finding reported previously for the OX26 TfRMAb (Bickel et al, 1994). It is difficult to detect the distribution of the TfRMAb into the post-vascular space of brain with IHC. The sharp contrast between the IHC signal over the brain endothelium vs. the post-vascular brain parenchyma arises from the nearly 1,000-fold difference in the volume of the brain capillary intra-endothelial space, which is 0.8 uL/Gram (Gjedde and Christensen, 1984), and the post-vascular space of brain, which is 700 uL/Gram (Pardridge, 2022b). As the TfRMAb passes out of the very small endothelial volume to enter the post-vascular volume of brain, the concentration of the antibody is diluted nearly 1,000-fold rendering it difficult to detect the antibody microscopically in the post-vascular brain. The detection of antibody accumulation in brain is possible with light microscopy in cases where the antibody is sequestered within focal regions of the post-vascular volume of brain such as amyloid plaques. This focal sequestration produces high local concentrations of the antibody in the post-vascular space of brain.

The hypothesis of Yu et al (2011) that high affinity TfRMAbs undergo minimal exocytosis into brain ISF ignores conflicting data, such as early work with carotid arterial infusion followed by capillary depletion and light microscopic emulsion autoradiography, which showed that the high affinity OX26 MAb undergoes rapid exocytosis into brain ISF at a rate comparable to holo-Tf (Skarlatos et al, 1995). If there was minimal exocytosis of a TfRMAb into brain ISF, then the high brain uptake of the high affinity TfRMAb could not be observed. Sequestration of the TfRMAb within the intra-endothelial volume, which is 1,000-fold smaller than the post-vascular volume of brain, would produce only background levels of brain uptake of the TfRMAb (Pardridge, 2021). TfRMAbs, which have been genetically engineered for low affinity, do not produce higher brain uptake. Affinity dematuration of complementarity determining regions (CDRs) of the OX26 antibody produced a variant, designated OX26-174, with low affinity for the TfR and a KD of 174 nM (Thom et al, 2018). However, no specific brain uptake of the OX26-174 variant was observed in the rat (Chang et al, 2021). Affinity dematuration produced a variant, designated OX26-76, with a low affinity for the rat TfR and a KD of 76 nM (Thom et al, 2018). The brain uptake of the OX26-76 was compared to the brain uptake of the wild type OX26 antibody, designated OX26-5, which had high affinity for the TfRMAb and a KD of 5 nM (Bonvicini et al, 2022). The brain uptake in the rat of the OX26-5 was 10-fold higher than the brain uptake of the OX26-76, which was at the background level (Bonvicini et al, 2022). The low affinity variants of the OX26 antibody were produced by mutations of amino acids within the CDRs of the variable regions of the OX26 antibody (Thom et al, 2018). No primary source of the amino acid sequences of the OX26 CDRs is reported, although the CDR sequences used are identical to that originally reported by Li et al (1999). A high level of brain uptake of a high affinity, bivalent VNAR-hFc TfRMAb is observed in the mouse following the IV injection of 1.9 mg/kg of the TfRMAb (Stocki et al, 2021), which parallels earlier reports on the brain uptake of a high affinity TfRMAb (Boado et al, 2009; Pardridge et al, 2018; Sonoda et al, 2018).

Developers of TfRMAb BBB delivery agents also propose that a monovalent TfRMAb is preferred over a bivalent TfRMAb. It is hypothesized that the bivalent TfRMAb triggers clustering of the TfR within the brain endothelium, which leads to triage of the complex to the lysosomal compartment and decreased TfR recycling to the membrane, and decreased TfR on the luminal endothelial membrane (Niewoehner et al, 2014). This hypothesis is based either on cell culture studies or on *in vivo* work where very high injection doses (ID), 50 mg/kg, of a high affinity TfRMAb is administered (Bien-Ly et al, 2014; Yu et al, 2014). With respect to the cell culture experiments, proponents of the intracellular TfR clustering hypothesis (Ullman et al, 2020; Morrison et al, 2023) cite cell culture work with a TfRMAb-avidin fusion protein (Ng et al, 2002; 2006) or a polyvalent form of Tf (Marsh et al, 1995). Owing to oligomerization caused by the avidin domain, the TfRMAb-avidin fusion protein forms a 400 kDa tetravalent complex (Ng et al, 2002; Boado et al, 2008), and is not representative of a bivalent TfRMAb. The polyvalent Tf used to demonstrate TfR clustering was an aggregate of Tf_10_ decamer (Marsh et al, 1995), and is not a physiologic ligand for the TfR. With respect to increased TfR degradation in brain following administration of a high affinity bivalent TfRMAb, this was observed following the IV injection of a very high ID of 50 mg/kg of the TfRMAb in the mouse (Bien-Ly et al, 2014; Yu et al, 2014). The IV administration of an ID of 50 mg/kg of a high affinity TfRMAb is a toxic dose, and is 50-fold greater than a therapeutic dose of a TfRMAb, which is 1 mg/kg as discussed below. Following the administration of 3–4 mg/kg of a high affinity TfRMAb, there is no suppression of the TfR either at the luminal membrane of the brain endothelium, or of the TfR concentration in brain parenchyma. A fusion protein of the high affinity bivalent chimeric 8D3 antibody and glial derived neurotrophic factor (GDNF) was administered IV to mice at an ID of 4 mg/kg/week for 12 weeks (Zhou et al, 2011a). The BBB permeability-surface area (PS) product of the TfRMAb-GDNF fusion protein, which is a measure of the abundance of the TfR on the brain endothelial luminal membrane, was unchanged at the end of 12 weeks of chronic dosing relative to the PS product for the fusion protein observed at the start of the chronic dosing study (Zhou et al, 2011a). Chronic dosing of 3 mg/kg every other day for 4 weeks of the high affinity bivalent chimeric 8D3 antibody to mice produced no change in the levels of either the TfR or iron in brain (Castellanos et al, 2020). Similarly, a 1.9 mg/kg IV dose of the high affinity, bivalent TXB2-hFc bivalent VNAR TfRMAb caused no decrease in the concentration of the TfR in brain (Stocki et al, 2021).

If high affinity bivalent TfRMAbs were sequestered within the brain capillary endothelium *in vivo*, as hypothesized by Yu et al (2011) and Niewoehner et al (2014), then the TfRMAb would not engage brain cells beyond the BBB, and it would not be possible to produce CNS pharmacologic effects with these antibodies. However, therapeutic effects in multiple mouse models of CNS disease were reported over 10 years ago with fusion proteins derived from a high affinity, bivalent TfRMAb. A TfRMAb-GDNF fusion protein was administered every other day at an ID of 1 mg/kg for 3 weeks via IV injection in a 6-hydroxydopamine model of PD in the mouse, and this treatment resulted in a 272% increase in striatal tyrosine hydroxylase (TH) enzyme activity that correlated with a 45% reduction in abnormal motor behavior induced by either apomorphine or amphetamine (Fu et al, 2010). The tumor necrosis factor receptor (TNFR) ECD, the active domain in etanercept, was fused to the carboxyl terminus of each heavy chain of the high affinity bivalent TfRMAb, and mice with experimental PD were treated every other day for 3 weeks with IV injections of saline, 1 mg/kg etanercept, or 1 mg/kg of the TfRMAb-TNFR fusion protein (Zhou et al, 2011b). Treatment with the TfRMAb-TNFR fusion protein produced a 130% increase in striatal TH enzyme activity, which correlated with a 75% reduction in abnormal motor behavior induced by apomorphine or amphetamine (Zhou et al, 2011b). Conversely, treatment with etanercept had no effect on striatal TH enzyme activity or motor function, as etanercept does not cross the BBB. The adult MPSI (Hurler) mouse was treated with a fusion protein of IDUA and the high affinity bivalent TfRMAb (Boado et al, 2011). The mice were treated twice weekly for 8 weeks with 1 mg/kg of the TfRMAb-IDUA fusion protein, and this treatment caused a 73% decrease in lysosomal inclusion bodies in the brain of the MPSI mice (Boado et al, 2011). High affinity bivalent TfRMAb fusion proteins were also neuroprotective in models of acute neural disease such as stroke. Experimental ischemia was induced in mice with a transient reversible middle cerebral artery occlusion (MCAO). Delayed IV treatment with 1 mg/kg of the TfRMAb-TNFR fusion protein caused a 45% reduction in cortical stroke volume, which correlated with a 48% reduction in neural deficit (Sumbria et al, 2012). Conversely, delayed treatment with 1 mg/kg etanercept IV had no therapeutic effect (Sumbria et al, 2012), because etanercept does not cross the BBB, and the BBB is intact in the early hours after stroke, when neuroprotection is still possible (Pardridge, 2022b). The TfRMAb-GDNF fusion protein was also neuroprotective in the reversible MCAO model in mice (Sumbria et al, 2013b). Delayed treatment with an IV dose of 1 mg/kg of the TfRMAb-GDNF fusion protein produced a 30% reduction in cortical stroke volume, and the combined delayed treatment of 1 mg/kg of the TfRMAb-GDNF and 1 mg/kg of the TfRMAb-TNFR fusion proteins produced a 69% reduction in cortical stroke volume in the reversible MCAO model of stroke in mice (Sumbria et al, 2013b). A TfRMAb-AβScFv BSA reduced brain amyloid, without cerebral microhemorrhage, in APP-PS1 AD transgenic mice with either twice-weekly IV injections of 1 mg/kg of the BSA (Zhou et al, 2011c), or daily subcutaneous (SQ) injections of 5 mg/kg of the BSA (Sumbria et al, 2013a), as discussed below in Section 3.3.1. No first injection reactions were observed in any of the mice investigated in these acute and chronic treatment studies with the high affinity bivalent TfRMAb.

Therapeutic effects in mouse models of neural disease are produced with IV doses of 1 mg/kg or SQ doses of 5 mg/kg using fusion proteins derived from a high affinity bivalent TfRMAb. The doses used in these models are a log order of magnitude lower than the therapeutic doses, 30–50 mg/kg, proposed by developers of low affinity, monovalent TfRMAb delivery agents (Yu et al, 2011). The therapeutic effects in multiple models of neural disease of 1 mg/kg doses of the fusion proteins derived from the high affinity, bivalent TfRMAb are consistent with early work showing that high affinity bivalent TfRMAbs undergo rapid transcytosis across the BBB *in vivo* (Skarlatos et al, 1995). Similar therapeutic effects in CNS disease models may be produced with low affinity, monovalent TfRMAbs, but at the expense of an ID that is 10- to 50-fold higher than that used for a high affinity bivalent TfRMAb. The higher ID required for a low affinity TfRMAb may reduce the therapeutic index of the drug, and increase the potential for toxicity from chronic administration of large doses of a TfRMAb.

### 3.3 BBB penetrating bispecific antibodies for CNS disease

A tetravalent BSA is formed from 2 separate heavy chains (HC) and 2 separate light chains (LC). Expression of a BSA in a host cell that is transfected with 2 HC genes and 2 LC genes presents multiple problems with respect to correct pairing of hetero-dimeric HCs in parallel with pairing of each HC with the cognate LC. Several strategies have been developed to minimize the expression of homo-dimeric antibodies and to maximize expression of the appropriate hetero-dimer. With the knob-in-hole (KiH) strategy, mutations of amino acids in the CH3 region are introduced to maximize pairing of the hetero-dimeric HCs, along with the expression of a single LC used by both antibody arms of the BSA (Ridgway et al, 1996). Amino acid mutations in the CH3 region of each HC create a “knob” in the CH3 region of antibody A and a “hole” in the CH3 region of antibody B. Mis-pairing of HCs is still a problem with the KiH approach, which prompted the original developers of the KiH strategy to add the engineering of an inter-chain disulfide linker following the insertion of cysteine (Cys) residues near the carboxyl terminus of the CH3 region (Merchant et al, 1998). Given the challenges of the KiH approach with respect to suppression of formation of homo-dimers, an electrostatic steering (ESS) strategy was developed (Gunasekaran et al, 2010; Wang F. et al, 2020), where amino acids in the CH3 region were mutated to form salt bridges between the CH3 regions of the different HCs. For example, E356K/D399K mutations in the CH3 region of the HC of antibody A (HC-A) forms salt bridges with K439D/K409E mutations in the CH3 region of the HC of antibody B (HC-B). Proper pairing of the HC-A with the LC-A, in parallel with pairing of the HC-B and LC-B was favored by introduction of an additional HC-LC disulfide formed following HC-F126C and LC-S121C mutations (Wang F. et al, 2020).

The KiH or ESS strategies produce a BSA where both antibody A and antibody B are engineered in a monovalent format. This obligates the developer of such a BSA to the use of moderate affinity, monovalent antibodies at both arms of the BSA. A tetravalent BSA, where both antibody A and B are engineered in a high affinity bivalent format, is made possible when either antibody A or B is engineered in a single chain design. As described by Coloma and Morrison (1997), a single chain Fv (ScFv) form of antibody A is fused to the carboxyl terminus of the HC of a second antibody B. This ‘Morrison antibody’ approach produces a hetero-tetrameric BSA following the transfection of the host cell with a single HC-ScFv fusion gene and a single LC gene. ScFv antibodies can form multimers (Wu et al, 2001). Multimer formation can be minimized or eliminated by insertion of a long 25-amino acid linker between the VH and VL domains of the ScFv, in parallel with mutations that insert Cys residues within FR2 of the VH domain and within FR4 of the VL domain of the ScFv (Schanzer et al, 2011). This intra-chain/inter-domain disulfide in a ScFv antibody was described by Reiter et al (1995). In lieu of a ScFv antibody, a single chain Fab (scFab) antibody, or a single domain antibody can be used such as a camelid VHH nanobody or a shark VNAR antibody, as discussed below. Alternatively, a dual variable domain (DVD) BSA may be engineered where the VH of antibody A is fused to the amino terminus of the VH region of antibody B, and the VL of antibody A is fused to the amino terminus of the VL region of antibody B (Jakob et al, 2010). The carboxyl terminus of the VH of antibody B is fused to the CH1-hinge-CH2-CH3 HC constant region, and the carboxyl terminus of the VL of antibody B is fused to the constant region of the light chain (CL). One potential issue with the DVD BSA is steric hindrance in binding of the “inner” antibody B to its cognate antigen caused the presence of the “outer” antibody A, as discussed below. Yet another strategy to BSA engineering is the production of a Fcab BSA, where several non-continuous amino acids in the CH3 region of antibody A are mutated so as to create a new antibody B binding site within the Fc region of antibody A. In this format, antibody A targets antigen A at the classical VH and VL domains at the amino terminus of the BSA (Wozniak-Knopp et al, 2010).

#### 3.3.1 Bispecific antibodies for Alzheimer’s disease derived from anti-Abeta amyloid antibodies

**FIGURE 6:**
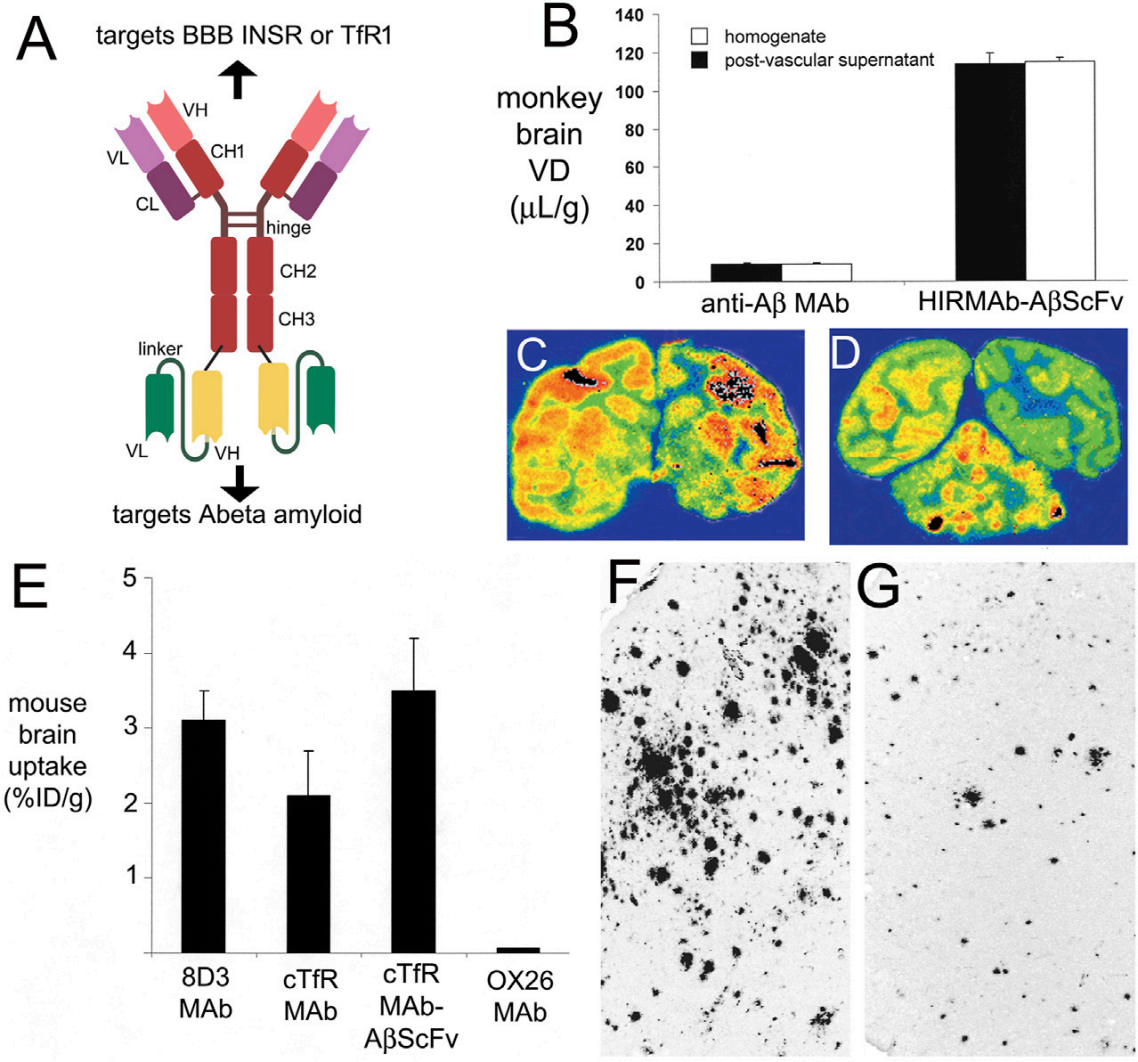
**(A)** Design of high affinity, tetravalent bispecific antibody (BSA) formed by fusion of a single chain Fv (ScFv) antibody, which targets the Aβ amyloid of AD, to the carboxyl terminus of the heavy chain of either a human/mouse chimeric antibody that targets the human insulin receptor (INSR) (Boado et al, 2007), and designated the HIRMAb-AβScFv BSA, or to the carboxyl terminus of the heavy chain of the rat/mouse chimeric 8D3 antibody that targets the mouse TfR1 (Boado et al, 2010), and designated the TfRMAb-AβScFv BSA. Drawing produced with Biorender.com. **(B)** The HIRMAb-AβScFv BSA was radio-iodinated, and the parent anti-Aβ MAb was tritiated and co-injected IV in the Rhesus monkey. The brain volume of distribution (VD) of the anti-Aβ MAb in either the total brain homogenate, or in the post-vascular supernatant, was 10 uL/Gram, which indicates the MAb does not cross the BBB and is confined to the brain plasma volume. Conversely, the brain VD of the HIRMAb-AβScFv BSA in either the homogenate or the post-vascular supernatant is high, 200 uL/Gram, which indicates the BSA crosses the BBB *in vivo* in the primate. The brain was removed 2 h after IV injection in the monkey and film autoradiography of coronal sections of midbrain **(C)** and hindbrain **(D)** shows global penetration of the [^125^I]-BSA into brain parenchyma with higher uptake in gray matter as compared to white matter. Panels B, C, and D reproduced with permission from Boado et al (2007). **(E)** Brain uptake, measured as % injection dose (ID)/G brain, of the rat 8D3 MAb against the mouse TfR, the rat/mouse 8D3-derived chimeric TfRMAb, the TfRMAb-AβScFv BSA, or the mouse OX26 MAb against the rat TfR, is measured at 60 min after IV administration in the mouse. Reproduced with permission from Boado et al (2010) **(F, G)** Inverted image of fluorescent microscopy of AD transgenic mouse brain immune-stained with the 6E10 MAb against Aβ fibrils following 12 weeks of treatment with either saline **(F)** or 5 mg/kg of daily subcutaneous administration of the TfRMAb-AβScFv BSA **(G)**. Panels F and G reproduced with permission from Sumbria et al (2013a).

**FIGURE 7:**
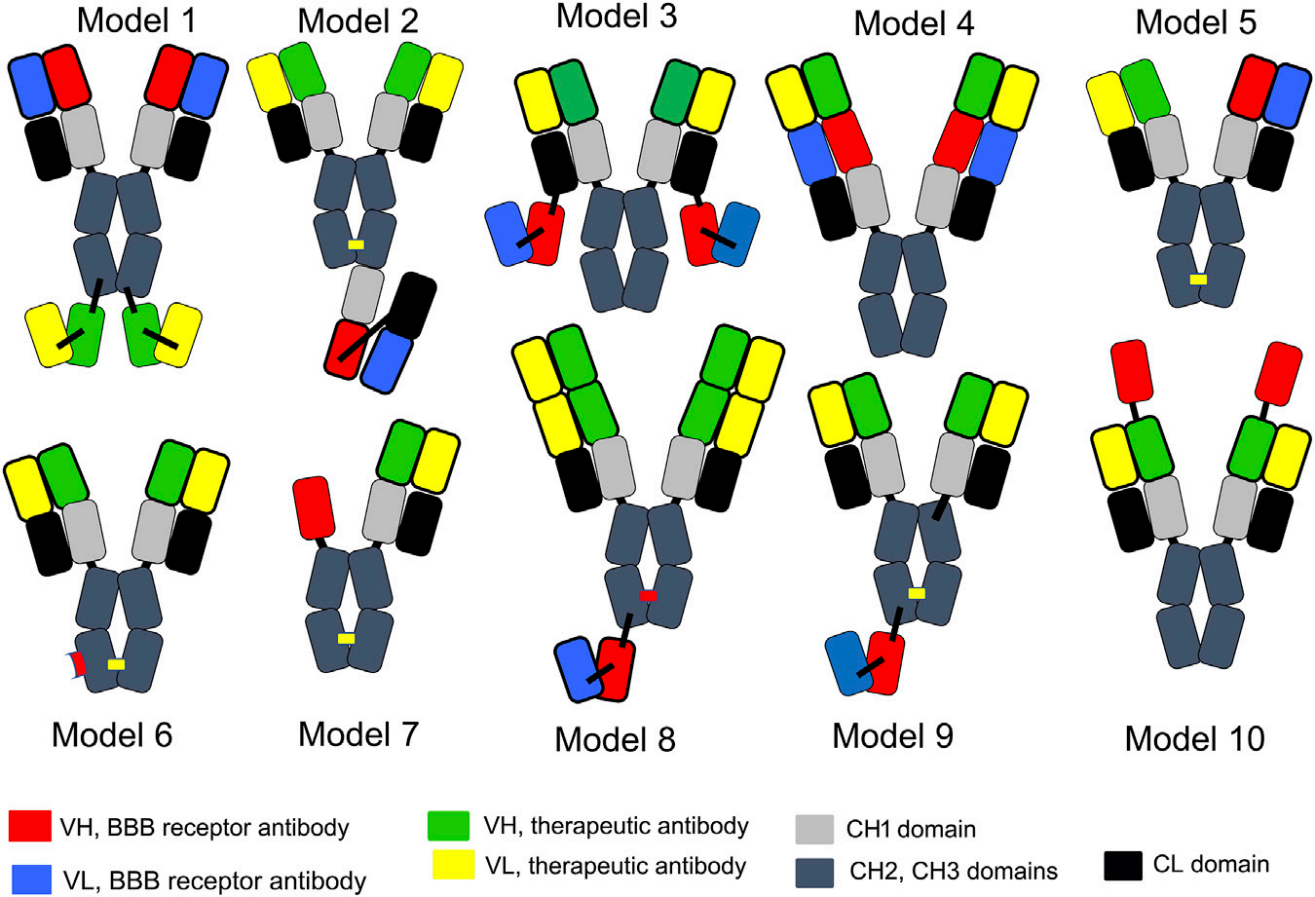
Ten models of BBB-penetrating bispecific antibodies (BSAs). Model 1 is a tetravalent BSA formed by fusion of a single chain Fv (ScFv) form of a therapeutic antibody to the carboxyl terminus of both heavy chains (HC) of the HIRMAb (Boado et al, 2007) or TfRMAb (Boado et al, 2010). Model 2 is a bivalent/monovalent BSA formed by fusion of a single chain Fab antibody against the TfR to the carboxyl terminus of one heavy chain of a therapeutic antibody, where knob-in-hole (KiH) technology is used to promote the formation of the hetero-dimer (Niewoehner et al, 2014). The KiH is depicted by the yellow bar in the CH3 regions of the half-antibodies. Model 3 is a tetravalent BSA formed by fusion of a ScFv antibody against the TfR to the carboxyl termini of both light chains (LC) of the therapeutic antibody (Hultqvist et al, 2017). Model 4 is a tetravalent dual variable domain BSA formed by fusion of the VH and VL of the therapeutic antibody to the amino terminus of the VH and VL of the TfRMAb (Karaoglu et al, 2018; Do et al, 2020). Model 5 is a dual monovalent BSA where the half-antibodies, with the cognate LC, against either the TfR or the therapeutic target, were produced separately in either bacteria (Yu et al, 2011) or CHO cells (Yu et al, 2014), purified separately, and paired by reductive annealing. Formation of hetero-dimers was promoted with KiH technology (yellow bar in CH3 region). Model 6 is monovalent/bivalent Fcab BSA where a TfR binding site is engineered in the CH3 region of a half-antibody against the therapeutic target. The other half antibody is directed only against the therapeutic target, and has no TfR binding site. The formation of the hetero-dimer of the half antibodies is promoted with KiH technology (Kariolis et al, 2020; Van Lengerich et al, 2023), as indicated by the yellow bar in the CH3 region. The TfR binding site engineered in the CH3 region of one half-antibody is depicted by the red bar in Model 6. Model 7 is a dual monovalent BSA formed by fusion of a camelid VHH nanobody against the TfR to the amino terminus of an Fc fragment, which is hetero-dimerized with a half antibody against the therapeutic target with KiH technology, as indicated by the yellow bar in the CH3 region (Wouters et al, 2022). Model 8 is monovalent/tetravalent BSA formed by fusion of a ScFv against the TfR to the carboxyl terminus of one HC of the therapeutic antibody, which has double VH domains in the HC and double VL domains in the light chain for tetravalent binding to the therapeutic target. Formation of the hetero-dimers is promoted by electrostatic steering (ESS), as indicated by the red bar in the CH3 region (Zhao et al, 2022). Model 9 is a monovalent/bivalent BSA formed by fusion of a ScFv antibody against the IGF1R to the carboxyl terminus of a therapeutic half-antibody, which is paired with the other half antibody lacking the ScFv using KiH technology, as indicated by the yellow bar in the CH3 region (Shin et al, 2022). Model 10 is a tetravalent BSA formed by fusion of a shark VNAR nanobody targeting the TfR to the amino terminus of the HC of the therapeutic antibody (Sehlin et al, 2020; Clarke et al, 2022).

The first BBB-penetrating BSAs were reported for the HIRMAb (Boado et al, 2007) and for the TfRMAb (Boado et al, 2010). An anti-Abeta amyloid antibody (AAA), targeting the amino terminal part of the Aβ^1-43^ peptide, was converted to a single chain Fv (ScFv) antibody with a 17 amino acid linker joining the VH and VL of the AAA, and this AβScFv was fused to the carboxyl terminus of each HC of the HIRMAb or TfRMAb, as depicted in Figure 6A. The ScFv derived tetravalent BSA is shown as Model 1 in Figure 7. The HIRMAb-AβScFv was radio-iodinated, and the intact 150 kDa AAA was tritiated with [^3^H]-N-succinimdyl proprionate, and the antibodies were co-injected IV in the Rhesus monkey. The brain VD of the [^3^H]-AAA was 10 uL/Gram (Figure 6B), which is equal to the brain blood volume, indicating the AAA alone does not cross the BBB. In contrast the brain uptake of the [^125^I]-HIRMAb-AβScFv BSA was enhanced to produce a VD of 120 uL/Gram (Figure 6B). Film autoradiography of the primate brain removed at 2 h after IV injection showed global penetration of the brain by the BSA with higher uptake in gray matter as compared to white matter (Figures 6C, D). The HIRMAb-AβScFv retained high affinity binding to the HIR ECD with a KD of 1.0 ± 0.1 nM, as compared to the KD of the HIR antibody, 0.53 ± 0.02 nM (Boado et al, 2007). The HIRMAb-AβScFv BSA caused disaggregation of Abeta amyloid fibrils *in vitro* and *in vivo* in the APP_swe_/PS1dE9 AD transgenic mouse, also known as the APP-PS1 mouse. A 20 pmol dose of the HIRMAb-AβScFv BSA, or saline, was injected into the cortex or hippocampus of the AD mouse and the brain amyloid plaque was quantified 48 h later with confocal microscopy following brain staining with thioflavin-S, which binds mature amyloid plaques. BSA treatment caused a 39% reduction in mature plaque compared to saline (Boado et al, 2007). The APP-PS1 AD mice could not be treated with the HIRMAb-AβScFv BSA via the IV route, because the HIRMAb does not recognize the murine insulin receptor (Zhou et al, 2012). To generate an anti-amyloid BSA that penetrates the BBB of the mouse following IV administration, the anti-Abeta ScFv was fused to the carboxyl terminal of the HCs of the 8D3 chimeric TfRMAb (Boado et al, 2010). The 8D3 chimeric TfRMAb is designated the cTfRMAb and the BSA formed from the cTfRMAb and the AβScFv is designated cTfRMAb-AβScFv. The cTfRMAb antibody and the cTfRMAb-AβScFv BSA were radio-labeled with the [^125^I]-Bolton-Hunter reagent, and injected IV in control mice. The brain uptake of the cTfRMAb-AβScFv BSA was 3% ID/Gram, which is comparable to the brain uptake in the mouse of either the 8D3 MAb or the engineered cTfRMAb (Figure 6E). In contrast, the brain uptake in the mouse of the OX26 MAb against the rat TfR is very low, as this antibody is entrapped in the brain plasma volume without BBB penetration (Figure 6E). OX26 does not cross the mouse BBB because the OX26 antibody is specific for the rat TfR and does not recognize the mouse TfR (Lee et al, 2000). The effect of chronic treatment with the cTfRMAb-AβScFv BSA on brain amyloid was examined in APP-PS1 AD transgenic mice (Zhou et al, 2011c; Sumbria et al, 2013a). In the first study, 12-month old APP-PS1 mice were treated for 12 weeks with twice-weekly injections of either saline or 1 mg/kg IV of the cTfRMAb-AβScFv BSA (Zhou et al, 2011c). Treatment caused a 40% decrease in immunoreactive Aβ^1-42^ in brain with no change in plasma Aβ^1-42^. Prussian blue histochemistry showed no cerebral microhemorrhage (Zhou et al, 2011c). In a second study, 12-month old APP-PS1 AD mice were treated daily for 12 weeks with either saline or the cTfRMAb-AβScFv BSA at dose of 5 mg/kg administered subcutaneously (SQ). The BSA is bioavailable following SQ administration, as the 24-h plasma area under the concentration curve (AUC) of a cTfRMAb fusion protein following a SQ injection in the mouse is comparable to the 1-h plasma AUC after an IV administration (Sumbria et al, 2013c). Following 12 weeks of treatment of the AD mice, the mature amyloid plaque was quantified with confocal microscopy following staining of cortex and hippocampus with thioflavin-S, and the amyloid fibrils was quantified with confocal microscopy following immune staining of the cortex and hippocampus with the 6E10 MAb (Sumbria et al, 2013a). The amyloid fibrils were reduced 56%–61% and the amyloid mature plaque was reduced 43%–48%. The 6E10 immune staining of the cortex of the AD mice treated with either saline or the cTfRMAb-AβScFv BSA is shown in Figures 6F, G, respectively. The disaggregation of amyloid plaque in the AD mouse caused by chronic treatment is evidence that the high affinity bivalent TfRMAb arm of the BSA enables RMT across the BBB followed by engagement of the therapeutic target in brain by the AAA arm of the BSA (Sumbria et al, 2013a). Prussian blue histochemistry of brain showed no cerebral microhemorrhage following 12 weeks of daily treatment with the 5 mg/kg SQ dose of the cTfRMAb-AβScFv BSA (Sumbria et al, 2013a).

A BSA targeting the TfR and the Abeta amyloid was engineered by fusion of a single chain Fab (scFab) antibody derived from a MAb against either the mouse or human TfR to the carboxyl terminus of the heavy chain of the mAb31 AAA, which is a humanized antibody against the Abeta amyloid peptide (Niewoehner et al, 2014). The scFab against the TfR was fused either to a single HC, designated sFab, or to both HCs, designated dFab, of the mAb31. The sFab was a moderate affinity monovalent TfRMAb, while the dFab was a high affinity bivalent TfRMAb. The affinity of the dFab binding to the TfR was 5-9-fold higher than the affinity of the sFab binding to the TfR. Based on cell culture studies, or *in vivo* uptake in mice at high IDs, 13–17 mg/kg, which saturate the TfR for a high affinity TfRMAb, it was concluded that a monovalent form of the antibody arm targeting the TfR was preferred over a bivalent form (Niewoehner et al, 2014). The brain concentration in mice of the sFab was 2.6 nM at 8 h after the IV administration of a 13 mg/kg dose of the sFab antibody (Niewoehner et al, 2014), which is equivalent to a low brain uptake of 0.03% ID/Gram brain. The BSA was engineered where the monovalent scFab, targeting the TfR, was fused to the carboxyl terminus of a single HC of the mAb31, using KiH technology, as depicted in Model 2 of Figure 7. The BSA formed from the mAb31 AAA and the scFab against the human TfR is also known as trontinemab, or RO7126209 (Alzforum, 2023a), and is the first BBB penetrating BSA to enter clinical trials. Following completion of a trial in healthy volunteers (NCT04023994), trontinemab is now being tested in an ascending dose clinical trial in subjects with AD (NCT04639050). Trontinemab represents a re-engineering of the AAA, gantenerumab. Gantenerumab was originally developed for AD (Bohrmann et al, 2012), but failed in clinical trials (Alzforum, 2023b).

A BSA targeting the Abeta amyloid and the TfR was engineered, where a scFv form of the 8D3 TfRMAb was fused with an 11 amino acid linker to the carboxyl terminus of each LC of the AAA, mAb158 (Hultqvist et al, 2017), as shown as Model 3 in Figure 7. The mAb158 is the mouse precursor to the humanized form, BAN2401, now known as lecanemab, as discussed in section 2.1.3.1. The 8D3scFv was fused to the carboxyl terminus of the LC rather than the HC, as it was hypothesized that fusion of the scFv to the light chain would eliminate bivalent binding of the 2 TfRMAb arms of the BSA to the TfR homo-dimer (Hultqvist et al, 2017). No supporting data was provided for the monovalent binding hypothesis other than a reduction in affinity measured by ELISA. However, the EC50 of binding of the 8D3scFv domain of the mAb158-8D3scFv BSA was still in the high affinity range, 8 nM (Hultqvist et al, 2017). The antigen used in the ELISA was the mouse TfR ECD, which includes the receptor stalk domain that forms a disulfide linked homo-dimer between 2 TfR units. Fusion of the 8D3scFv to the C-terminus of the light chain of the mAb158 antibody would produce monovalent binding to the TfR only if this fusion engineered a separation of the paired scFvs that was either too wide, or too narrow, to enable bivalent binding to each receptor of the TfR homo-dimer. The distance between antibody arms that enables bivalent binding to 2 epitopes is fairly wide, and only a 2-fold variation in avidity is observed over a separation range of 7–20 nm for an IgG1 antibody (Jendroszek and Kjaergaard, 2021). This distance between 2 arms of an antibody engaged in bivalent binding of 2 epitopes encompasses the 9–10 nm width of the extracellular domain of the TfR1 dimer (Fuchs et al, 1998). Therefore, the high affinity binding of the mAb158-8D3scFv to the mouse TfR likely arises from bivalent binding of the BSA to the TfR homo-dimer.

The brain uptake of the lecanemab precursor, mAb158, in the mouse is low, 0.028% ID/Gram (Hultqvist et al, 2107), which represents antibody sequestration in the brain plasma volume. However, the brain uptake of the mAb31-8D3scFv BSA was high, 2.2% ID/Gram (Hultqvist et al, 2107). The brain uptake of this high affinity mAb31-8D3scFv BSA was saturated by an IV dose of 10 mg/kg of the 8D3 TfRMAb (Hultqvist et al, 2107). Lecanemab preferentially binds protofibrils, which are soluble Abeta oligomers (Cummings et al, 2023). The mAb31-8D3scFv BSA retains high affinity binding to Aβ oligomers, and the engagement by the mAb31-8D3scFv BSA of Abeta protofibrils in the brain of the ArcSwe AD transgenic mouse was demonstrated following the IV administration of 5 mg/kg of this BBB-penetrating BSA (Rofo et al, 2022).

An alternative form of a tetravalent BSA is the DVD format shown as Model 4 in Figure 7. A DVD BSA was engineered where 1 antibody arm targeted the mouse TfR, and the other antibody arm was derived from the sequence of the 3D6 AAA (Karaoglu et al, 2018). Either short, five to six amino acid, or long, 11–12 amino acid, linkers were placed between the 2 VH and 2 VL domains. With the DVD format, there is an ‘inner’ antibody and an ‘outer’ antibody as shown in Model 4 (Figure 7). The outer antibody is expected to cause steric hindrance of the binding of the inner antibody to the target antigen, which may be minimized by the insertion of a longer linker between the 2 variable domains of the HC and LC. When the TfRMAb is used as the inner antibody, the affinity of DVD binding to the mouse TfR is reduced compared to the parent TfRMAb (Karaoglu et al, 2018). The model TfR-3D6 BSA was designated anti-Aβ-LS-anti-TfR1 (AB221), where the 3D6 anti-Aβ antibody is placed in the outer domain and the anti-TfR1 is placed in the inner domain. The Aβ-LS-anti-TfR1 (AB221) DVD had a 10-fold reduced affinity for the TfR, as compared to the parent TfRMAb, but was still in the high affinity range with an EC50 of 1.2 nM (Karaoglu et al, 2018). This DVD was not tested in an AD mouse model. A similar DVD BSA was engineered with the 8D3 TfRMAb and the 13C3 MAb against Aβ protofibrils (Do et al, 2020). BSAs were engineered in the aglycosylated form with the N289A mutation to reduce effector function of the antibody (Do et al, 2020). The KD of binding to the mouse TfR was 0.33 nM when the 8D3 antibody was placed in the outer domain of the DVD, and designated TBTI2; conversely, the KD of binding to the mouse TfR was 15 nM when the 8D3 antibody was placed in the inner domain of the DVD, which was designated TBTI1 (Do et al, 2020). The affinity of the TBTI1 antibody for the TfR was further reduced by affinity dematuration of CDRs, based on the assumption that a reduced affinity TfRMAb was the preferred agent for BSA delivery to brain. These mutated DVDs were designated TBTI3, TBTI4, TBTI5, and TBTI6, which had a KD of binding to the TfR of 6.9 nM, 9.0 nM, 29 nM, and 20 nM, respectively (Do et al, 2020). Of the 6 TBTI BSAs, 4 have an affinity for the TfR that would be considered high affinity, where KD = 0.4–9 nM, and 2 have an affinity that would be considered moderate affinity, where KD = 20–29 nM. The BSA brain concentration was measured at 24 h after an IV injection of ∼15 mg/kg of the 6 TBTI BSAs, TBTI1-6 (Do et al, 2020). Analysis of the brain uptake data for these 6 BSAs shows an inverse relationship between KD and brain concentration (R = 0.75). The lower the affinity of the DVD for the TfR, i.e., the higher the KD of binding, then the lower the brain concentration of the BSA. The study shows that affinity dematuration to reduce the affinity of the TfRMAb for the TfR had an adverse effect on brain uptake of the BSA.

#### 3.3.2 Bispecific antibodies for Alzheimer’s disease derived from anti-BACE1 antibodies

The beta-site amyloid precursor protein cleaving enzyme 1 (BACE1) mediates the near carboxyl terminal cleavage of the amyloid precursor protein (APP) to generate monomeric forms of the Aβ^1-40/43^ peptide, which aggregates to form the amyloid plaque in AD (Hampel et al, 2021). A major objective within the pharmaceutical industry is the development of small molecule BACE1 inhibitors. However, there are no approved small molecule BACE1 inhibitors as the clinical trials in AD of the lead candidates have failed owing to toxicity or poor drug penetration of the BBB (Pardridge, 2020). An alternative strategy is the development of an anti-BACE1 MAb. However, these agents do not cross the BBB (Yadav et al, 2017), and must be re-engineered as a BBB-penetrating BSA. A BSA of an anti-human BACE1 MAb and a TfRMAb was produced using KiH technology (Yu et al, 2011). Both the BACE1 antibody and the TfR antibody of the BSA were engineered as monovalent arms as shown by Model 5 of Figure 7. Both TfRMAb and BACE1 half antibodies were expressed as aglycosylated antibodies in *E. coli*, and were annealed to form the hetero-dimer following purification. Engineering the TfRMAb as a monovalent antibody produced the expected reduction in affinity for the TfR. A high affinity bivalent TfRMAb, specific for the mouse TfR, was engineered with a KD of 1.7 nM. However, when this high affinity TfRMAb, designated TfR^A^, was engineered as the monovalent arm of the BSA, the affinity of the TfR^A^ arm of the BSA for the mouse TfR was reduced with a KD of 45 nM (Yu et al, 2011). The high affinity bivalent TfRMAb, TfR^A^, was affinity dematured to produce TfR^B^, TfR^C^, and TfR^D^, and these variants bound to the TfR with a KD of 6.9 nM, 65 nM, and 111 nM, respectively. The brain uptake of the TfR^A^ and TfR^D^ was compared following an injection of antibody dose of 4 mg/kg, which was considered a “low dose” and an antibody dose of 20–50 mg/kg, which was considered a “therapeutic dose.” Since a dose of 4 mg/kg fully saturates the brain uptake of a high affinity TfRMAb (Lee et al, 2000), the brain uptake was expectedly higher at doses of 20–50 mg/kg for the low affinity TfRMAb (Yu et al, 2011). The low affinity TfR^D^ antibody, with a KD of 111 nM of binding to the TfR, was said to be the preferred BBB delivery agent (Yu et al, 2011). A 50 mg/kg dose of the anti-TfR/BACE1 BSA reduced brain Aβ^1-40^ levels in mouse brain, but at this very high ID, the anti-BACE1 alone also reduced brain Aβ^1-40^ (Yu et al, 2011). The same group engineered an anti-TfR/BACE1 BSA specific for the human TfR, and the TfRMAb cross reacted with the cynomolgus monkey TfR (Yu et al, 2014). The N297G mutation was used to reduce effector function of the aglycosylated BSA (Yu et al, 2014). The TfRMAb and BACE1 antibodies were separately expressed in Chinese hamster ovary (CHO) cells, and the two half-antibodies were combined to form the hetero-dimer following reductive annealing (Yu et al, 2014). Two variants of the BSA were engineered and designated TfR^1^/BACE1 and TfR^2^/BACE1. The TfR^1^/BACE1 had a KD for the human and primate TfR of 10 nM and 37 nM, whereas TfR^2^/BACE1 had a KD for the human and primate TfR of 270 nM and 810 nM, respectively (Yu et al, 2014). The higher affinity BSA, which has a KD of 10 nM for the human TfR, was said to be the preferred delivery agent, and not the low affinity BSA, which had a KD of 270 nM (Yu et al, 2014). A 20 mg/kg dose of the high affinity BSA was observed to cause a decrease in brain TfR in the mouse, but did not reduce the brain TfR in the primate (Yu et al, 2014). The study of Yu et al (2014) proposes the optimal KD of the TfRMAb for the TfR is 10 nM, as opposed to the prior study of Yu et al (2011), which proposed the optimal KD of the TfRMAb was 10-fold lower, 111 nM.

A low affinity TfRMAb/BACE1 BSA was engineered (Kariolis et al, 2020), which employed the Fcab technology (Wozniak-Knopp et al, 2010). To reduce effector function, the L234A/L235A (LALA) mutations (Wines et al, 2000) were inserted in the upper hinge/CH2 region of the heavy chains (Kariolis et al, 2020). The bivalent BACE1 antibody was engineered with a TfR binding site in the CH3 region of one of the BACE1 antibody heavy chains as depicted by Model 6 in Figure 7. This low affinity TfR binding site was produced by mutating 9 discontinuous amino acids in the distal part of the CH3 region of one of the BACE1 antibody heavy chains. The hetero-dimer was formed with KiH technology (Kariolis et al, 2020). This TfRMAb/BACE1 BSA had a low affinity for the human TfR, KD = 120 nM, and a very low affinity for the cynomolgus monkey TfR, KD = 1,900 nM (Kariolis et al, 2020). These KD values were determined by surface plasmon resonance using a recombinant form of the apical domain of the human TfR. Given the marked loss of affinity of this BSA for the cynomolgus monkey TfR, it is not clear if safety pharmacology/toxicology studies in the primate can be extrapolated to humans (Kariolis et al, 2020).

A TfRMAb camelid VHH nanobody/BACE1 BSA was engineered following immunization of an alpaca with either the human TfR ECD, or the mouse TfR ECD, and isolation of a VHH nanobody specific for the human TfR, designated Nb188, or the mouse TfR, designated Nb62 (Wouters et al, 2022). The 15 kDa Nb62 VHH was fused to the carboxyl terminus of the light chain of a BACE1 murine antibody, designated 1A11WT, to form a tetravalent BSA with a bivalent TfRMAb, and this BSA was designated 1A11WT-2xNb62. The affinity of the monovalent Nb62 for the mouse TfR was moderate with a KD of 18 nM. However, the affinity of the bivalent 1A11WT-2xNb62 for the mouse TfR was high with a KD of 2.4 nM. The affinity of the monovalent Nb188 for the human TfR was high with a KD of 6.4 nM (Wouters et al, 2022). Working under the assumption that a monovalent TfRMAb was preferred over a bivalent TfRMAb, the Nb188 was fused to the amino terminus of a human Fc, which was paired with humanized anti-BACE1 half antibody to generate a BSA designated 1A11AM-Nb188, and this BSA is represented by Model 7 in Figure 7. The 2 chains of the 1A11AM-Nb188 BSA were paired to form the hetero-dimer with the Bipod method (Nesspor et al, 2020). The affinity of the 1A11AM-Nb188 BSA for the human TfR was not reported. This BSA was tested in transgenic mice where the apical domain of the human TfR was knocked in to replace the apical domain of the mouse TfR. Administration of a high dose, 20 mg/kg, but not a low dose, 2 mg/kg, of the this BSA resulted in a modest reduction in the Aβ^1-40^ levels in brain of the transgenic mice (Wouters et al, 2022). The lack of reduction of brain Aβ^1-40^ levels with the 2 mg/kg dose is attributed to the low affinity of this BSA that is monovalent for the TfR.

#### 3.3.3 Bispecific antibodies for Alzheimer’s disease derived from TREM2 agonist antibodies

The triggering receptor for myeloid cells 2 (TREM2) is expressed on microglial cells, and TREM2 activation leads to phagocytosis of oligomeric Aβ fibrils (Griciuc et al, 2019). Therefore, a TREM2 agonist MAb could be therapeutic in AD, should the antibody be made transportable through the BBB. A TREM2 agonist antibody, AL002 (Wang S. et al, 2020), is currently in clinical trials for AD with a primary endpoint of improvement in the CDR-SB score (NCT04592874). AL002 was taken to clinical trial based on the rationale that this therapeutic antibody crossed the BBB. The model used by the AL002 drug developers (Wang S. et al, 2020) was the same model used by the aducanumab drug developers (Sevigny et al, 2016), which is that higher amounts of the antibody were detected in brain homogenate when the injection dose was increased. However, higher plasma concentrations of the antibody are produced at higher injection doses. If the antibody is confined to the brain plasma volume, visualized in Figure 3, and if the plasma compartment is incompletely cleared by intra-cardiac saline infusion, then measurable amounts of the antibody will be detected in a homogenate of brain, without any passage of the antibody across the BBB. The maximal plasma concentration of the AL002 antibody was 59, 351, and 1,014 ug/mL after the intra-peritoneal (IP) administration of 5, 20, and 60 mg/kg; the corresponding maximal brain concentration of the antibody was 1.8, 7.6, and 23.8 ng/mg protein in the mouse (Wang S. et al, 2020). Assuming 100 mg protein per Gram brain (Dunlop et al, 1994), the brain/plasma concentration ratio, which is the brain VD of AL002, is two to three uL/Gram, which is about 10% of the brain plasma volume in the mouse (Section 2.2.2). It is possible that the AL002 measured in brain homogenate represents contamination of the brain by plasma due to a washout of only 90% of the brain plasma. One could measure the albumin concentration in brain homogenate to test for residual plasma in the brain homogenate, but this is not done.

Working on the view that a TREM2 agonist antibody needs to be re-engineered to enable BBB penetration, a BSA derived from a TfRMAb and a TREM2 agonist antibody was engineered (Zhao et al, 2022). A TREM2 agonist ScFv antibody was isolated by screening a human ScFv library with the murine TREM2 ECD, and a lead ScFv was identified (Zhao et al, 2022). The VH and VL of several candidates were grafted on to human IgG1, resulting in the identification of a lead agonist antibody, Ab18. However, the agonist activity of this antibody was not optimal. Therefore, a DVD of the Ab18 was engineered, as shown in Model 8 of Figure 7. The DVD design increased the potency of the TREM2 antibody as the EC50 of agonist activity was reduced to 0.42 nM following engineering of this antibody that has tetravalent binding to TREM2. To enable BBB penetration of the TREM2 tetravalent DVD, a ScFv form of an antibody against the mouse TfR was fused to the carboxyl terminus of one heavy chain of the TREM2 DVD, as shown in Model 8 of Figure 7. Owing to this BSA design, the 2 heavy chains arise from different genes, and the formation of the hetero-dimeric heavy chains was enhanced with electrostatic steering (ESS) technology (Zhao et al, 2022). The origin of the sequence of the ScFv against the mouse TfR is not given (Zhao et al, 2022). A monovalent format of the TfRMAb was used, as it was believed that a bivalent TfRMAb is entrapped in the endothelium without entry into brain parenchyma based on prior hypotheses reported in the literature (Yu et al, 2011; Niewoehner et al, 2014; Yu et al, 2014). However, the hypothesis that low affinity, monovalent TfRMAbs are preferred BBB delivery agents is based on questionable premises, and the exclusion of contrary data, as reviewed in Section 3.2.4. The use of a monovalent format of the TfRMAb obligates the drug developer to a log order increase in injection dose, relative to a high affinity TfRMAb. Indeed, mouse treatment studies with the TREM2 DVD-TfRScFv employed weekly injections of 20 mg/kg in 5XFAD transgenic mice (Zhao et al, 2022). This treatment resulted in a decrease in brain Aβ amyloid in parallel with an increase in synaptic density. This finding of a repair of dystrophic neurites following local clearance of amyloid plaque replicates the effects of an intra-cerebral injection of an AAA into the brain of an AD transgenic mouse (Lombardo et al, 2003).

A BBB-penetrating TREM2 agonist antibody suitable for clinical trials has recently been described (Van Lengerich et al, 2023). The design of this BSA, designated ATV:TREM2, where ATV = antibody transport vehicle, replicates Model 6 of Figure 7. A MAb against human TREM2 was produced following immunization of rodents with the human TREM2 ECD. This MAb against hTREM2 bound with high affinity to human TREM2, KD = 2.0 nM, but did not bind murine TREM2 (Van Lengerich et al, 2023). Using Fcab technology, a monovalent TfR binding site was engineered in the CH3 region of one heavy chain of the anti-hTREM2, and the 2 half-antibodies were hetero-dimerized with the KiH method. To reduce effector function, the LALA mutations were inserted in the upper hinge/CH2 region of the heavy chains (Van Lengerich et al, 2023). This project extends the use of low affinity TfRMAbs to new lows, as the KD of binding of ATV:TREM2 to the human TfR was 1,430 nM (Van Lengerich et al, 2023), or 3 log orders of magnitude lower than a conventional high affinity TfRMAb. Bioactivity of the ATV:TREM2 was assessed in a double transgenic mouse, hTREM2 tg; TfR^mu/hu^, where the apical domain of the murine TfR was replaced with the apical domain of the human TfR (Van Lengerich et al, 2023). The intravenous administration of 10 mg/kg of the ATV:TREM2 BSA in the transgenic mice produced a peak brain concentration of 3 nM (Van Lengerich et al, 2023), which corresponds to a brain antibody uptake of 0.15% ID/Gram. A similar, if not identical, ATV:TREM2 BSA, is currently in phase one clinical trials in healthy volunteers (NCT05450549). This trial is being performed in the Netherlands, and not in the U.S. The FDA placed a clinical hold on the IND application for the ATV:TREM2 in the U.S. owing to concerns about preclinical toxicology related to this therapeutic product (Alzforum, 2023c).

#### 3.3.4 Bispecific antibodies for Parkinson’s disease derived from anti-α-synuclein antibodies

PD is associated with the formation of intracellular α-synuclein (SYN) aggregates (Volpicelli-Daley et al, 2011) and an antibody that disaggregates SYN inclusions could be therapeutic for PD. One anti-SYN antibody is the Syn-02 antibody, which binds SYN aggregates, but not soluble SYN (Vaikath et al, 2015). A ScFv form of the 8D3 antibody to the mouse TfR was fused to the carboxyl terminus of each light chain of an engineered form of the Syn-02 antibody, and this BSA was designated AbSynO2-scFv8D3 (Roshanbin et al, 2022), which conforms to Model 3 in Figure 7. The biologic activity of the AbSynO2-scFv8D3 BSA was tested in the L61 mouse, which is derived from a transgenic mouse line that over-produces human SYN aggregates (Rockenstein et al, 2002). The L61/SYN mice were treated with 10 mg/kg of either the Syn-02 antibody alone or the AbSynO2-scFv8D3 BSA on days 1, 2, and 4, and euthanized on day 5. This short course produced a modest decrease in brain SYN oligomers (Roshanbin et al, 2022). It is anticipated that a longer course of treatment will produce further decreases in brain SYN oligomers with such BBB-penetrating BSAs directed against α-SYN.

A BBB-penetrating BSA directed against α-synuclein was engineered with an anti-IGF1R MAb, designated Grabody B (Shin et al, 2022). A human ScFv library was screened with the human IGF1R ECD, and affinity maturation of initial candidates produced Grabody B, which had a high affinity for the IGF1R across species (Shin et al, 2022). The KD in surface plasmon resonance studies was in the 1–6 nM range, and the EC50 in ELISAs was in the 0.12–0.4 nM range. In parallel, a MAb against human SYN, designated M30103, was produced (Shin et al, 2022). A BSA, designated B30104, was formed by fusion of the IGF1R ScFv to the carboxyl terminus of one heavy chain of the M30103 antibody, and the 2 half-antibodies were hetero-dimerized with KiH technology. This BSA conforms to Model 9 of Figure 7. Brain B30104 concentration was linearly related to the intravenous ID and increased to 30 nM as the ID was increased from 10 to 60 mg/kg in the rat (Shin et al, 2022). A brain BSA concentration of 30 nM at an ID of 60 mg/kg corresponds to a brain uptake of 0.08 %ID/Gram. A mouse model of PD was produced following the intra-cerebral injection of pre-formed fibrils (PFF) of human SYN (Volpicelli-Daley et al, 2011). Treatment of mice was started 1 week following the PFF injection and continued with weekly intra-peritoneal (IP) injections of 15–18 mg/kg of either the M30103 anti-Syn antibody or the B30104 BSA for 6 months. Treatment of the mice with the BSA caused a reduction in SYN aggregates in brain. However, at these high injection does, administration of the M30101 antibody alone also caused a reduction in SYN aggregates in brain (Shin et al, 2022). Re-engineering of this BSA as a tetravalent BSA that has bivalent binding to both the IGF1R and to SYN aggregates would increase the affinity of the BSA for the IGF1R, which would enable the administration of a lower ID, e.g., 1 mg/kg. This reduced dose of the tetravalent BSA may lower SYN aggregates in brain, whereas there may no effect on SYN aggregates following the administration of a 1 mg/kg dose of the M30301 antibody alone.

#### 3.3.5 Bispecific antibodies for Parkinson’s disease derived from TrkB agonist antibodies

A potential treatment of neurodegeneration is the delivery to brain of neurotrophic factors, such as brain derived neurotrophic factor (BDNF). Over 25 years ago, a clinical trial in amyotrophic lateral sclerosis (ALS) investigated the therapeutic effects of chronic treatment with BDNF, where the neurotrophin was administered by SQ injection. The clinical trial failed (BDNF Study Group, 1999), as BDNF does not cross the BBB (Pardridge, 2022b). The BDNF receptor is trkB. An alternative approach to neurotrophin therapy of neurodegeneration is the delivery to brain of a trkB agonist antibody. One such trkB agonist antibody is the 29D7 antibody, which binds with high affinity to both the murine and human trkB in an ELISA with an EC50 = 10–100 pM, and exerts trkB agonist activity in cell culture assays (Qian et al, 2006). The BBB transport of the 29D7 trkB agonist antibody was enabled by the re-engineering of this antibody as a BBB-penetrating BSA. A single chain, single domain shark VNAR antibody against the TfR was fused to both heavy chains of the 29D7 antibody (Clarke et al, 2022). The anti-TfR VNAR is designated TXB4, which was derived from an early VNAR, designated TXB2 (Stocki et al, 2021). TXB2 was converted to TXB4 by mutagenesis of the CDR3 of the VNAR (Clarke et al, 2022). TXB2 was first used to engineer a BSA, where the TXB2 was fused to the amino terminus of both heavy chains of the bapineuzumab anti-amyloid antibody (Sehlin et al, 2020), and this BSA corresponds to Model 10 in Figure 7. The TXB4-derived BSA was engineered by placement of the TXB4 VNAR at the amino terminus of both heavy chains of the 29D7 antibody, and this format was designated the HC2N BSA. Owing to concerns about steric hindrance of binding to trkB of the inner 29D7 antibody by the outer VNAR TfR antibody, a second BSA was engineered where the VNAR was placed between the hinge region and CH1 of both heavy chains of the 29D7 antibody, and this format was designated the HV2N BSA. It is not clear why the VNAR against the TfR was not fused to the carboxyl termini of the 29D7 antibody, as this would eliminate steric hindrance of binding of the amino terminal end of the BSA to the trkB receptor. The HC2N BSA had a 3-fold higher affinity for the mouse or human TfR1 as compared to HV2N, although both BSAs bound the TfR with high affinity and an EC50 of 0.7–2.3 nM in the TfR1 ELISA (Clarke et al, 2022). The HV2N BSA was more active in a trkB bio-assay with an EC50 of 0.91 nM, as compared to the EC50 of the HC2N BSA, which had an EC50 of 6.9 nM. Therefore, subsequent *in vivo* studies were performed with the HV2N BSA, designated the TXB4-TrkB (Clarke et al, 2022). The therapeutic effect of the TXB4-TrkB BSA was examined in experimental PD produced by the intra-striatal injection of the neurotoxin, 6-hydroxydopamine. Mice were treated with SQ injections of either saline or 2.5–5 mg/kg of the TXB4-TrkB BSA at days −1 and +7 relative to toxin administration. This dose of toxin produces a partial lesion of the nigral striatal tract in mice, and the number of cells immunoreactive for tyrosine hydroxylase (TH) was reduced 27% in the substantia nigra on the lesioned side compared to the contralateral side in the saline treated mice. There was only a 3% reduction in TH-immunoreactive cells in the mice treated with the TXB4-TrkB BSA (Clarke et al, 2022). Given the design of this study, where the TXB4-TrkB BSA was administered 24 h before the toxin injection, it is not clear if delayed treatment with the TXB4-TrkB BSA is neuroprotective in experimental PD, but this issue can be resolved with future investigations.

## 4 Pharmacokinetics and toxicology

There is little information on the plasma pharmacokinetics (PK) of a BBB-penetrating BSA compared to the plasma PK of the original therapeutic antibody. The plasma clearance of a therapeutic antibody that does not target an RMT system is slow, e.g., the plasma half-life (T_1/2_) of donanemab is 10.5 days at an IV dose of 10 mg/kg (Lowe et al, 2021). Fusion of a therapeutic antibody to a transporting antibody that targets an RMT system such as the IR, TfR, or IGF1R, is expected to cause an increase in plasma clearance of the BSA, relative to the therapeutic antibody, as demonstrated for the PK of an Abeta therapeutic antibody and the HIRMAb-AβScFv BSA (Boado et al, 2007). If the plasma clearance of the BSA was increased 5- to 10-fold, compared to the native antibody, then the plasma T_1/2_ would be reduced to 1–2 days. In this case, the optimal route of administration of a BBB-penetrating BSA for treatment of a CNS disease may be the SQ route with twice weekly administration, as opposed to once- or twice-monthly IV infusions of a therapeutic antibody. The optimal therapeutic dose of a BBB-penetrating BSA derived from a high affinity transporting antibody may be 3 mg/kg administered via SQ injection.

Doses of a BBB-penetrating BSA above 3 mg/kg may have toxicologic side effects. Anemia associated with reduced reticulocytes was produced in primates following the twice weekly IV infusions of a high affinity bivalent TfRMAb at a dose of 30 mg/kg, but not at doses of 3–10 mg/kg (Pardridge, et al, 2018). This study replicates the early findings in the mouse describing anemia induced by the administration of a low affinity TfRMAb (Couch et al, 2013). The anemia was partially ameliorated in a FcγR knockout mouse, and the effect of TfRMAb on blood counts was caused by both antibody-dependent cytotoxicity (ADCC) and complement medicated cytotoxicity (CDC) (Couch et al, 2013). The IgG-mediated effector functions are reduced with the L234A/L235A (LALA) mutations (Wines et al, 2000), which reduce ADCC, and the P329G (PG) mutation, which reduces CDC, and these mutations are designated LALA-PG (Lo et al, 2017). The LALA mutations reduce IgG binding to the FcγR, but not the neonatal Fc receptor, FcRn (Wines et al, 2000). TfRMAb-derived fusion proteins may not express the same effector function as the parent antibody. A high affinity bivalent TfRMAb induced CDC, but not ADCC in human cells; however, following fusion of the IDS lysosomal enzyme to this TfRMAb, no CDC was observed with the TfRMAb-IDS fusion protein (Yamamoto et al, 2021). FcγRs are also expressed in the human brain, on both microglia and neurons (Fuller et al, 2014). The effect of IgG administration on effector function in the CNS is not observed with therapeutic antibodies, because these agents do not cross the BBB. However, a TfRMAb that activates effector function would cross the BBB and access FcγRs within the brain. Chronic administration of a TfRMAb to Rhesus monkeys induced astrogliosis and microglial activation (Pardridge et al, 2018). This antibody was not mutated to eliminate effector function. Nevertheless, it is important to perform neuropathologic exams of brain in primates following chronic administration of a new BBB-penetrating BSA early in the course of drug development.

## 5 Conclusion

This review advances 2 themes. First, therapeutic antibodies do not cross the intact BBB. Second, therapeutic antibodies can be re-engineered as BBB-penetrating bispecific antibodies (BSAs), which target endogenous receptor-mediated transport (RMT) systems within the BBB. The conclusion that therapeutic antibodies do not cross the BBB would seem to be at odds with the FDA approval of therapeutic antibodies for the treatment of multiple sclerosis (MS), glioma, and Alzheimer’s disease (AD). However, as discussed in Section 2.1, the therapeutic antibodies that have been approved by the FDA for the CNS either have a site of action within the blood compartment, as with the case of antibodies for MS or glioma, or enter the brain following antibody-induced BBB disruption, as with anti-amyloid antibodies (AAA) for AD. The BBB disruption is associated with the amyloid related imaging abnormalities (ARIA) as detected with MRI in subjects with AD following the administration of AAAs. The absence of brain uptake of a therapeutic antibody that does not target an endogenous RMT system at the BBB, and confinement of the therapeutic antibody to the brain plasma volume, is shown in Figure 6B for the monkey and in Figure 6E for the mouse. The data showing entrapment of a therapeutic antibody within the brain plasma volume is discussed in section 2.1.3.1 for aducanumab or bapineuzumab, and in section 3.3.3 for AL002, a TREM2 agonist antibody. These data confirm early reports on the lack of brain uptake of immunoglobulin G (Kobayashi et al, 1989), or of a [^111^In]-labeled MAb (Eigenmann et al, 2017). An illustration of the lack of BBB transport of a therapeutic antibody is the study showing that BBB disruption induced by focused ultrasound-microbubble treatment is required to detect brain uptake of a therapeutic antibody (Janowicz et al, 2019).

A BBB-penetrating BSA is formed by fusion of a therapeutic antibody and a transporting antibody. The latter undergoes RMT across the BBB following the antibody binding to an endogenous peptide receptor on the BBB. These peptide receptors include the insulin receptor (IR), transferrin receptor (TfR), insulin-like growth factor receptor (IGFR), or leptin receptor (LEPR). Since the first description of a BBB penetrating BSA that targeted the human IR (Boado et al, 2007) or mouse TfR (Boado et al, 2010), a broad array of BSAs have been engineered for BBB delivery, as outlined in Figure 7. The engineering of these BSAs is derived from 2 different approaches. In the first approach, the host cell is transfected with a single heavy chain (HC) gene and a single light chain (LC) gene followed by the expression of a tetravalent BSA that binds bivalently with both the therapeutic target and the BBB target. This is possible by fusion of a single chain Fv (ScFv) antibody A to the carboxyl terminus of the HC or LC of antibody B (Model 1 for HC fusion and Model 3 for LC fusion, Figure 7), as originally described by Coloma and Morrison (1997), and refined by Schanzer et al (2011). Alternatively, the ScFv can be replaced by a single chain Fab (scFab) antibody (Hust et al, 2007), a single domain camelid VHH or a single domain shark VNAR. Tetravalent BSAs may also be engineered where the variable domains of both antibodies are placed at the amino terminus of the BSA, as in the case of a dual variable domain BSA (Model 4, Figure 7), or fusion of a single chain antibody A to the amino terminus of antibody B (Model 10 Figure 7). In the case of Models 1, 3, 4, and 10, the therapeutic antibody and the transporting antibody are both engineered as a bivalent antibody. In the second approach, the host cell is transfected with 2 HC genes and a single LC gene, and the HC hetero-dimer formation is promoted with knob-in-hole (KiH) technology (Models 2, 5, 6, 7, and 9, Figure 7), or with electrostatic steering (ESS) technology (Model 8, Figure 7). For the Model 5 BSA, the host cell must be transfected with either a single LC that is shared by both the therapeutic antibody and the transporting antibody, or with 2 LC genes. When separate LC genes are expressed, then the host cell is transfected with 4 genes (2 HC genes, 2 LC genes), and the pairing of the cognate HC and LC for the therapeutic antibody and the transporting antibody is promoted by engineering new disulfide bridges between the HC and LC (Wang F. et al, 2020). In the case of Models 2, 5, 6, 7, 8, and 9, the therapeutic antibody is engineered as a bivalent antibody, and the transporting antibody is engineered as a monovalent antibody.

The use of a monovalent transporting antibody is based on the hypothesis that a monovalent antibody is preferred over a bivalent antibody, and this hypothesis is derived from use of a TfRMAb transporting antibody. A high affinity, bivalent TfRMAb is said to be sequestered within the brain endothelium leading to reduced exocytosis into brain ISF, and diminished BBB transport (Yu et al, 2011; Niewoehner et al, 2014; Ullman et al, 2020). However, if the bivalent TfRMAb was sequestered within the endothelium, then the high brain uptake of a high affinity bivalent TfRMAb observed in the mouse (Figure 6E) or the primate (Pardridge et al, 2018) would not be possible. Sequestration of the antibody within the brain endothelium would produce a background level of brain uptake of the antibody because the intracellular volume of the endothelium is 1,000-fold smaller than the post-vascular volume of brain (Pardridge, 2021). The hypothesis that a bivalent TfRMAb is not effectively transcytosed through the BBB ignores the therapeutic effects in multiple models of neural disease, including PD, AD, MPSI, and stoke, which are produced following the administration of low doses, 1 mg/kg, of fusion proteins derived from a high affinity, bivalent TfRMAb (Section 3.2.4). The use of a low affinity, monovalent TfRMAb requires the administration of doses of the BSA, e.g., 15–50 mg/kg (Yu et al, 2011; Kariolois et al, 2020), that are a log order greater than the therapeutic dose used for a high affinity bivalent TfRMAb, e.g., 1 mg/kg. The engineering of a BSA with reduced affinity for binding to the BBB RMT system obligates the drug developer to the administration of a higher dosage of the BSA. Such high doses may reduce the therapeutic index of the drug, and produce unwanted side effects from BSA treatment.
